# Lineage-Specific Proteomic Signatures in the *Mycobacterium tuberculosis* Complex Reveal Differential Abundance of Proteins Involved in Virulence, DNA Repair, CRISPR-Cas, Bioenergetics and Lipid Metabolism

**DOI:** 10.3389/fmicb.2020.550760

**Published:** 2020-09-22

**Authors:** Solomon Abebe Yimer, Shewit Kalayou, Håvard Homberset, Alemayehu Godana Birhanu, Tahira Riaz, Ephrem Debebe Zegeye, Timo Lutter, Markos Abebe, Carol Holm-Hansen, Abraham Aseffa, Tone Tønjum

**Affiliations:** ^1^Unit for Genome Dynamics, Department of Microbiology, University of Oslo, Oslo, Norway; ^2^Coalition for Epidemic Preparedness Innovations, Oslo, Norway; ^3^Division of Laboratory Medicine, Department of Microbiology, Oslo University Hospital, Oslo, Norway; ^4^International Centre of Insect Physiology and Ecology, Nairobi, Kenya; ^5^Institute of Biotechnology, Addis Ababa University, Addis Ababa, Ethiopia; ^6^NORCE Norwegian Research Centre AS, Centre for Applied Biotechnology, Bergen, Norway; ^7^Armauer Hansen Research Institute, Addis Ababa, Ethiopia; ^8^Division of Infection Control and Environmental Health, Norwegian Institute of Public Health, Oslo, Norway

**Keywords:** *Mycobacterium tuberculosis*, lineage 7, proteomics, DNA repair, ESX-3 secretion system, virulence, Ethiopia

## Abstract

Despite the discovery of the tubercle bacillus more than 130 years ago, its physiology and the mechanisms of virulence are still not fully understood. A comprehensive analysis of the proteomes of members of the human-adapted *Mycobacterium tuberculosis* complex (MTBC) lineages 3, 4, 5, and 7 was conducted to better understand the evolution of virulence and other physiological characteristics. Unique and shared proteomic signatures in these modern, pre-modern and ancient MTBC lineages, as deduced from quantitative bioinformatics analyses of high-resolution mass spectrometry data, were delineated. The main proteomic findings were verified by using immunoblotting. In addition, analysis of multiple genome alignment of members of the same lineages was performed. Label-free peptide quantification of whole cells from MTBC lineages 3, 4, 5, and 7 yielded a total of 38,346 unique peptides derived from 3092 proteins, representing 77% coverage of the predicted proteome. MTBC lineage-specific differential expression was observed for 539 proteins. Lineage 7 exhibited a markedly reduced abundance of proteins involved in DNA repair, type VII ESX-3 and ESX-1 secretion systems, lipid metabolism and inorganic phosphate uptake, and an increased abundance of proteins involved in alternative pathways of the TCA cycle and the CRISPR-Cas system as compared to the other lineages. Lineages 3 and 4 exhibited a higher abundance of proteins involved in virulence, DNA repair, drug resistance and other metabolic pathways. The high throughput analysis of the MTBC proteome by super-resolution mass spectrometry provided an insight into the differential expression of proteins between MTBC lineages 3, 4, 5, and 7 that may explain the slow growth and reduced virulence, metabolic flexibility, and the ability to survive under adverse growth conditions of lineage 7.

## Introduction

Despite the discovery of *Mycobacterium tuberculosis* (Mtb) more than 130 years ago, Mtb physiology and the mechanisms of virulence are still not fully understood. More than 10.4 million individuals develop active tuberculosis (TB) globally every year, causing 1.3 million deaths annually (World Health Organization [WHO], 2019). The high morbidity and mortality associated with disease caused by Mtb reflects challenges in detecting and diagnosing TB at early stages, as well as poor therapeutic options. The urgent need for improved diagnostics, chemotherapeutics and vaccines for TB is well recognized. A better understanding of the biology of Mtb may expedite the development of the new and innovative tools in demand.

Mtb is a facultative intracellular organism that can adapt to the human host and exerts virulence and immune surveillance (Barth et al., 2013). A better understanding of the mechanisms by Mtb evades the host defense system may contribute to the development of novel therapeutics for TB.

Even though the major cause of TB in humans is Mtb, there are also other members of the human-adapted *Mycobacterium tuberculosis complex* (MTBC) that are responsible for TB. These include the five human-adapted lineages representing *M. tuberculosis* sensu stricto (lineages 1–4 and lineage 7), and lineage 5 and 6 which are commonly named as *M. africanum*. It also included some of the nine animal-adapted lineages already described earlier (O’Neill et al., 2019; Bottai et al., 2020; Coscolla et al., 2020). In addition, a recent study revealed the discovery of a human-adapted Mtb lineage 8 (Ngabonziza et al., 2020). Therefore, high quality genomic and proteomic data from human-adapted MTBC lineages-infected cells are warranted.

The MTBC lineages are classified as ancestral and “modern.” The genomes of the “modern” lineages 2 (Beijing), 3 (CAS/Delhi) and 4 (Haarlem) have undergone a large deletion known as TbD1. Lineages 2, 3, and 4 are associated with major TB epidemics (Firdessa et al., 2013; Yimer et al., 2013; Bottai et al., 2020) and have higher transmission rates than lineages 1 (Indo Oceanic), 5 (*M. africanum* 1) and 6 (*M. africanum* 2) (Chacon-Salinas et al., 2005), and lineage 7 (Yimer et al., 2015). Lineage 3 has a higher anti-inflammatory phenotype than lineage 4 (Portevin et al., 2011). “Modern” Mtb lineages induce less of an early inflammatory response than lineages 1 and 6 (Portevin et al., 2011). Strains of *M. africanum* (lineage 6) develop drug resistance less frequently (Tientcheu et al., 2016), while lineage 2 (Beijing) acquires drug resistance more frequently (Nguyen et al., 2016). In order to elucidate unique and shared phenotypes and levels of virulence of MTBC lineages, high resolution proteomic data providing direct and highly detailed functional information about cellular metabolism is imperative (Kelkar et al., 2011; Jena et al., 2013; Bespyatykh et al., 2016; Jhingan et al., 2016; Banaei-Esfahani et al., 2017; Schubert et al., 2018).

As part of our ongoing TB research with strains from the Horn of Africa, several genomics and proteomics studies have been conducted to better understand the clinical relevance of Mtb lineage 7. Mtb lineage 7 strains appear to grow more slowly *in vitro* than other Mtb lineages (Yimer et al., 2015). Patients infected with Mtb lineage 7 were associated with long delays in seeking health care (Yimer et al., 2015). Mtb lineage 7 cells frequently carry a high number of mutations in genes involved in DNA repair, replication and recombination, carbohydrate transport and metabolism, and energy production and conversion (Yimer et al., 2016). Compared to H37Rv, Mtb lineage 7 exhibits reduced abundance and hypoacetylation of proteins involved in growth and virulence (Birhanu et al., 2017; Yimer et al., 2017). However, available genomic and proteomic data do not explain the observation that lineage 7 is associated with a high rate of TB transmission, despite an apparent slower rate of growth (Yimer et al., 2015). To address the unique features of different MTBC lineages, we undertook a comprehensive proteomic study of MTBC lineages 3, 4, 5, and 7, focusing on proteins relevant to lipid metabolism, virulence, DNA repair, oxidative phosphorylation, CRISPR-Cas systems and drug resistance.

The differential expression of 3092 MTBC proteins, covering 77% of the predicted MTBC proteome, were included in this study. Principal components analysis, pathway clustering and other bioinformatics methods were used to identify unique protein signatures and potentially lineage-specific protein-protein interactions. Proteins involved in the TCA cycle and CRISPR-Cas system were more abundant in lineage 7 than in other lineages. The significance and implications of the results for MTBC lineages metabolism and pathogenesis are discussed.

## Materials and Methods

### Cell Culture, Lysis and In-Gel Digestion

Strains from MTBC lineages 3 (ID: Sol 101), 4 (ID: FSP 471) and 5 and two strains from lineage 7 (ID: L735 and ID: L728) were cultivated and harvested as previously described (Yimer et al., 2017). The MTBC isolates were cultured on Middlebrook 7H10 agar plates in triplicate for 32 days, harvested, rinsed in PBS, and heat-inactivated (80°C for 90 min). Cell pellets were resuspended in lysis buffer; 10 mM Tris-HCl (pH 7.5), 2% SDS, 1 tablet per 50 mL EDTA-free Protease Inhibitor Cocktail (Sigma-Aldrich), 1 tablet per 10 mL PhosSTOP Phosphatase Inhibitor Cocktail (Roche). The samples were transferred to Lysing Matrix B tubes (Roche) and mechanically disrupted using a bead beater (MagNa Lyser, Roche Diagnostics). The lysates were clarified by centrifugation (15,000 × *g* for 15 min) at 21°C, and the supernatant was transferred to 2 mL screw-cap micro-tubes (Sarstedt, Nümbrecht, Germany).

Protein samples (100 μg) were precipitated overnight by dilution with four volumes of ice-cold acetone. Precipitated proteins were centrifuged at 15,000 × *g* for 15 min at 4°C. The supernatant was aspirated and the protein pellet air-dried. Protein pellets were re-solubilized in NuPAGE LDS sample buffer (4x) (Life Technologies, United States) and reduced using 10 mM DL-dithiothreitol. Solubilized samples were incubated for 10 min at 70°C and proteins size-separated by SDS-PAGE (1.0 mm, 4–12% NuPAGE Novex Bis-Tris SDS-PAGE gel [Life Technologies]) at 80 V for 5 min followed by 20 min at 200 V. Gels were stained with colloidal Coomassie blue according to the manufacturer’s instructions. Stained protein lanes were excised from the gel and cut into six pieces using a scalpel. The gel pieces were transferred to a micro-centrifuge tube and destained sequentially with 500 μl 50% propanol, 500 μl 100% propanol and then centrifuged at 600 rpm at room temperature. In-gel reduction, alkylation, and tryptic digestion were performed as previously described (Shevchenko et al., 2006), and customized for Mtb (Yimer et al., 2017).

Prior to alkylation, 100 μL of 10 mM DL-dithiothreitol in 100 mM ammonium bicarbonate (ABC) was added to the samples and incubated at 56°C for 1 h. Samples were alkylated in 55 mM iodoacetamide (Sigma-Aldrich, Cleveland, OH, United States) in 100 mM ABC for 1 h at room temperature in the dark. Gel pieces were washed sequentially with propanol and thereafter dehydrated under vacuum (Eppendorf, Speedvac concentrator 5301). Proteins were digested with sequencing-grade trypsin (1 μg trypsin/50 μg protein, Promega, WI, United States) for 16 h at 37°C in 50 mM ABC. Peptides were extracted from the gel by two-fold sequential treatment with 50% and 100% acetonitrile (ACN). Trypsin was inactivated by addition of 10% trifluoroacetic acid (TFA). Samples were dehydrated under vacuum as above and re-suspended in 0.05% TFA. Peptides were desalted using C18 stage tips (Rappsilber et al., 2003) with minor modifications (Yimer et al., 2017). Samples were resuspended in 20 μL 0.1% formic acid (FA), transferred to nano-liquid chromatography vials and stored at −20°C until further analysis.

### Liquid Chromatography and Mass Spectrometry

Mass spectrometry (MS) analysis was performed using a Q Exactive plus hybrid quadrupole-orbitrap instrument coupled with an EASY-nLC 1200 UHPLC system (Thermo-Fisher Scientific, Biberach, Germany). Samples (6 μL) were injected onto C_18_ Acclaim PepMap columns (3 μm particles, 25 cm × 75 μm ID), followed by PepMap RSLC C_18_ (2 μm particles), both from Thermo Fisher Scientific (Biberach, Germany). Separation was performed in a 65 min solvent gradient (0.1% FA in 80/20 acetonitrile/water to 0.1% FA) at 0.3 μL/min at 40°C. MS was performed in the data-dependent acquisition mode with automatic switching between MS and MS/MS scans of peptide precursors from 380 to 1500 m/z with a 3 × 10^6^ ion count target and a maximum injection time of 50 ms. The full MS scans were acquired at 70,000 resolution. The automatic gain control (AGC) target was 3 × 10^6^ ions with maximum injection time of 50 ms. The AGC target for MS/MS was 10^1^ and maximum injection time 100 ms with dynamic exclusion of 30 s.

### Protein Identification and Quantification

Raw tandem MS/MS data were analyzed using MaxQuant (MQ) version 1.4.0.5 (Cox and Mann, 2008) and the peptide database search engine Andromeda (Cox et al., 2011) to search the Mtb H37Rv reference proteome UP000001584 (3993 entries, accessed in July 2017, UniProt-proteome), reverse decoy databases (Elias and Gygi, 2007), and select laboratory contaminants. Enzyme specificity as trypsin without proline with a maximum of two missed cleavages and carbamidomethylation of cysteine as a fixed modification, was used for peptide identification. Acetylation of the N-terminus, conversion of N-terminal glutamine and glutamic acid to pyroglutamic acid, and oxidation of methionine were included as variable modifications. The first search for precursor ions was performed with a mass tolerance of 20 ppm for calibration, while 6 ppm was applied for the main search. For protein identification, at least 1 unique peptide was required per protein group. A minimum peptide length of 7 amino acids was required for identification. To increase the number of peptides that can be used for quantification, the “requantify” and “match between runs” features of MQ were utilized with a retention time alignment window of 3 min. The default global Label-free Quantification (LFQ) algorithm was enabled (Cox et al., 2014) with the minimum LFQ ratio count for the quantification of a protein set to 1. In addition, unique peptides were used for peptide quantification. The maximum false discovery rate (FDR) cutoff of 0.01 (1%) was set at both the peptide spectra matches and the protein group levels.

### Experimental Design

Protein samples (100 μg) were analyzed by SDS-PAGE. To reduce sample complexity and improve comprehensiveness of protein profile (Jafari et al., 2012), each biological experiment was fractionated into six gel bands, resulting in a total of 72 analytical runs (four lineages ^∗^ three biological experiments ^∗^ six fractions). Two categorical groupings were formed to represent (1) lineage identifier and (2) biological experiment ID. Analysis of quantitative changes in protein abundance was determined by the label-free algorithm.

### Bioinformatics and Statistical Analysis

Data filtration, transformation, normalization, visualization, exploratory analysis and statistical analysis were performed in the Perseus platform (Version 1.5.5.3) (Tyanova et al., 2016). LFQ intensities from «Protein Group» output of MQ were loaded into the Perseus software. Filters were applied to remove protein contaminants, reverse database identifications and proteins identified only by their modified site. LFQ intensity values were subjected to log2 transformation. Quantitative accuracy of LFQ intensities in the biological experiment was assessed by Pearson correlation coefficient and the distribution visualized by histogram (Supplementary File S1). Principal component analysis was performed on protein abundances to determine the inter-lineage variance. Venn diagrams were plotted in the Venn diagram tool^2^ (Heberle et al., 2015).

For statistical analysis, at least two valid LFQ intensities out of the three biological experiments were required. Missing values were imputed with random numbers from a normal distribution (parameters Width = 0.3, Shift = 1.8) (Hubner et al., 2010). Multiple-sample analysis of grouped biological replicates (permutation-based FDR cutoff of 0.01, S_0_ = 2.) was performed to identify significantly changed proteins across the different lineages. Welch’s *t*-test with an FDR value of 0.01 and S_0_ = 2 was applied to identify proteins when the abundance was significantly changed between any of two MTBC lineages.

**FIGURE 1:**
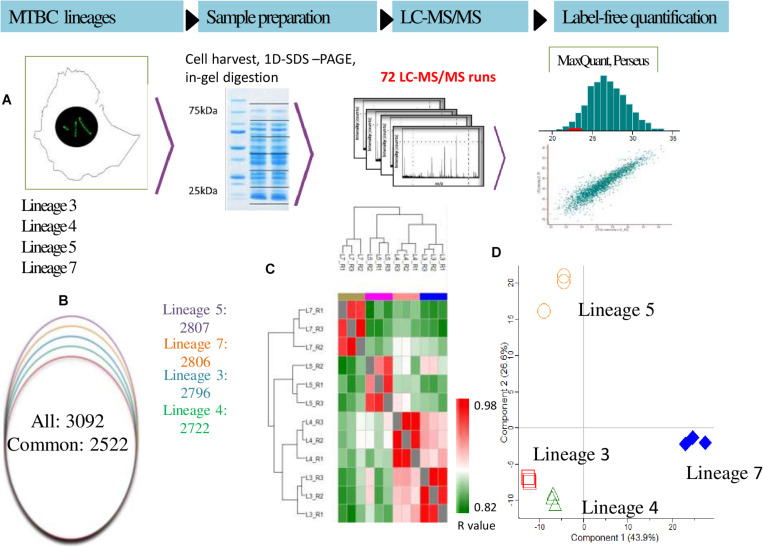
**(A–D)** Proteomics analysis of *M. tuberculosis* complex (MTBC) lineages. **(A)** Experimental workflow used in this study includes lysis, protein precipitation, tryptic digestion, peptide desalting, and ultra-high-performance liquid chromatography followed by mass spectrometry on a Q exactive plus mass spectrometer (Thermo-Fischer, Inc.). Label free quantification and the downstream statistical analysis were performed using MaxQuant and Perseus software, respectively. **(B)** Overlap of differentially abundant proteins in MTBC Lineages 3, 4, 5, and 7. **(C)** iBAQ-based ranking of protein abundances in various lineages. **(D)** Principal component analysis for protein abundance data from MTBC lineages. Samples were separated by lineage (component 1, 43.9% variance) and by biological groups (component 2, 26.6% variance). Only proteins identified in all samples were considered for the analysis. Data from three biological replicates are represented.

Analysis of GO annotation enrichment terms of the significant proteins were performed as previously described (Birhanu et al., 2017). Briefly, a two-tailed Fisher’s exact test was used to assess the significance of enrichment terms. Proteins assigned to enriched term categories (*p*-value <0.05) were grouped according to the Kyoto Encyclopedia of Genes and Genomes (KEGG) classification and Tuberculist’s protein functional category (Kapopoulou et al., 2011). To identify molecular complexes in the large protein interaction network, MCODE clustering algorithm based on Bader and Hogue (2003) was used. The complete experimental workflow applied including in-gel digestion image is depicted in Figure 1A.

The Search Tool for the Retrieval of Interacting Genes version 10.0 (Franceschini et al., 2013; STRING^3^) was used to interpret the biological relevance of DA proteins in terms of predicted protein-protein interaction networks. Predicted interaction networks were finally visualized using the Cytoscape software^4^ (Shannon et al., 2003).

### Multiple-Genome Alignment Analysis

GenBank files of MTBC L1, L3, L4 (including H37Rv), L5 and L6 were downloaded from NCBI. For lineage 2 (ID no. DA 3 and DA 10) and lineage 7 (ID no. L735 and L728), Illumina MiSeq raw data was quality assessed with FastQC, quality- and adapter-trimmed with BBDuk, *de novo* assembled with SPAdes, and contigs mapped against H37Rv with Bowtie2. Analysis of multi-genome alignment of all lineages focusing on CRISPR-Cas locus was performed using the Geneious Prime software (Geneious Prime 2020.1^4^).

### Data Handling

The mass spectrometry proteomics data have been deposited to the ProteomeXchange Consortium via the PRIDE (Perez-Riverol et al., 2019) partner repository with the dataset identifier PXD020383.

### Immunoblotting

A sample of 15 μg from each lysate was separated on a SDS-PAGE gel and transferred by blotting to a PVDF membrane. The membrane was blocked with 5% dry skim milk in TBS-T (10 mM Tris pH = 7.5, 150 mM NaCl, 0.05% TWEEN 20), incubated with ESAT-6 monoclonal antibody (11G4) from Thermo Fisher Scientific or GroEL2 mouse monoclonal antibody (BDI578) from OriGene diluted 1:1000 in TBS-T with 5% skim milk, and further incubated with HRP conjugated secondary antibodies (Santa Cruz SC-516102) diluted 1:5000 in TBS-T with 5% milk. Washing with TBS-T was done in between each step. The blot was photographed with luminescence detection using ECL^TM^ Prime (GE Healthcare) and ChemiDoc Touch (Bio-Rad Laboratories). A stain-free image (Bio-Rad Laboratories) of the blot was taken to ensure equal loading and transfer to membrane.

### Ethical Approval

The study obtained ethical approval from the Regional Committee for Medical Research Ethics in Eastern Norway (REK Øst) and the Ethiopian Science and Technology Ministry in Addis Ababa, Ethiopia. Written informed consent was obtained from the participants who provided the Mtb samples before the study was conducted.

## Results

### Protein Identification

A total of 38,346 unique MTBC peptides were identified, corresponding to 3,092 proteins that represent 77% coverage of the predicted MTBC lineage proteome. More than 81% (2522/3092) of the proteins were detected in all four lineages (Supplementary File S2). The spectral quality analysis from each biological replicate is presented in a histogram (Figure 1A). The total number of proteins identified in MTBC lineages 5, 7, 3, and 4 were 2807, 2806, 2796, and 2722, respectively (Figure 1B).

**FIGURE 2:**
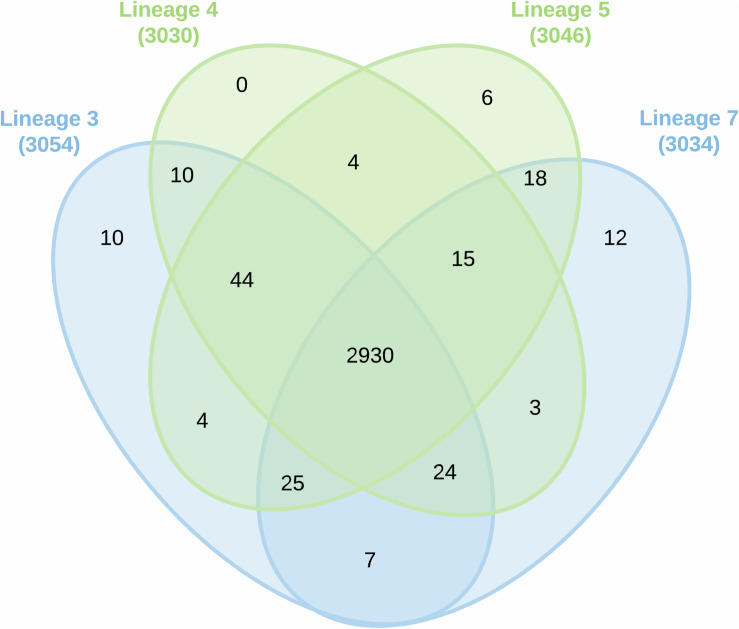
Venn diagram showing protein overlap among the various MTBC lineages of 3, 4, 5, and 7.

The proteomic data were analyzed using hierarchical clustering analysis (HCA) (Figure 1C) and principal component analysis (PCA) (Figure 1D). For HCA, LFQ intensities were used to calculate Pearson correlations (*R*-values) for each biological replicate (Figure 1C) and the resulting *R*-values were sufficiently high for reliable comparisons of protein abundances. Using proteins with 100% PCA valid values, the four lineages clustered together thus confirming data reproducibility. Considerable variation was detected among all the lineages (Figure 1D). Proteins expressed uniquely or in common between biological replicates and MTBC lineages are summarized in the Venn diagram (Figure 2).

### Dynamic Range of Protein Abundance

The iBAQ intensity data were used to rank the most abundant and least abundant proteins in each MTBC lineage. The 10 most abundant protein groups in lineage 7 included chaperones, kinase, acyl carrier and elongation factor, hemagglutinin and DNA ligases, and the least abundant proteins included oxidoreductases, ATP-binding protein, aminotransferase, nucleotide transferases and other uncharacterized proteins. The most abundant protein groups in lineage 4 comprised chaperones, acyl carrier, ESAT-6 like protein (ExsB), hydrolase, methyltransferase, and antioxidant proteins, and the least abundant protein groups included acyltransferase, nucleotide transferase, DNA-binding proteins and oxidoreductases. The iBAQ intensities in all proteomes covered four orders of magnitude (comparing the most and least abundant proteins) (Supplementary File S3).

**FIGURE 3:**
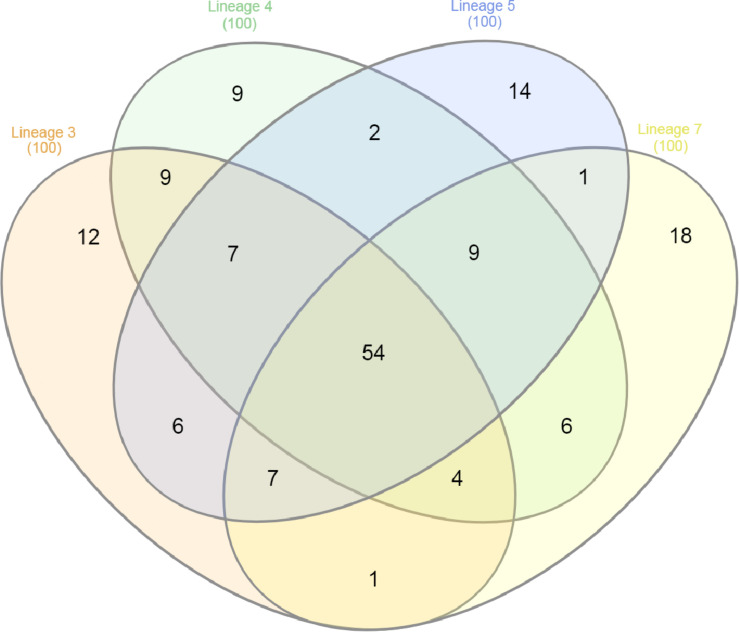
Venn diagram showing protein overlap among the top 100 most abundant proteins identified in the MTBC lineages of 3, 4, 5, and 7.

**FIGURE 4:**
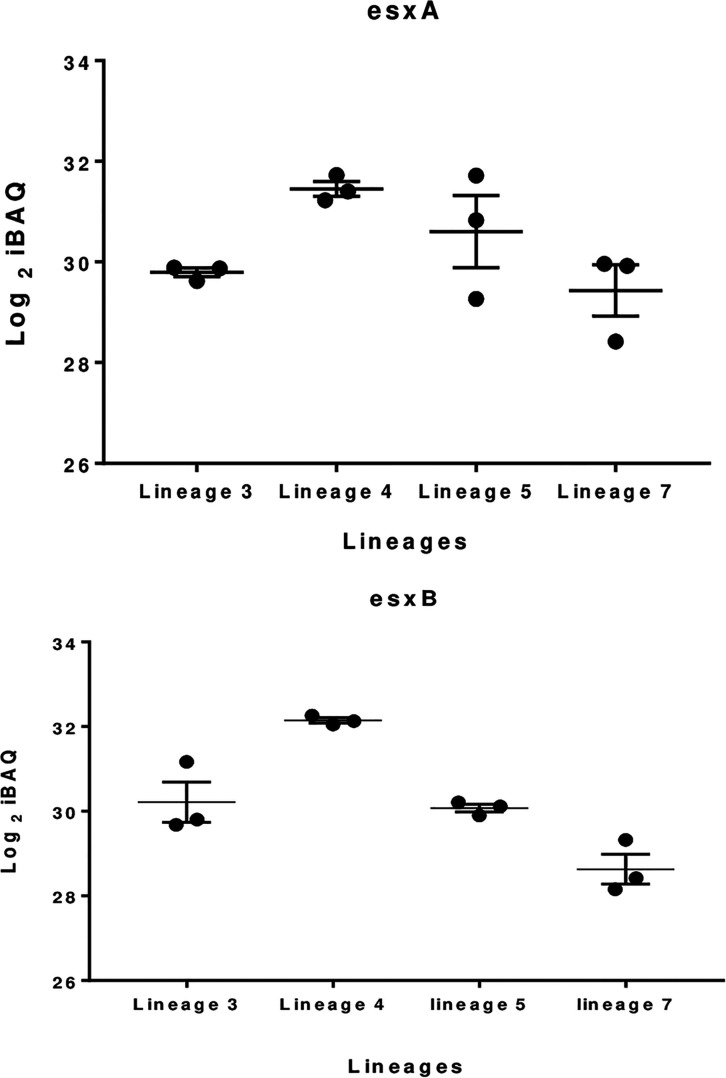
Boxplot showing difference in EsxA and EsxB proteins abundance in the various MTBC lineages.

The 100 most abundant proteins in each lineage were also analyzed; 9, 12, 14, and 18 proteins were unique to lineages 3, 4, 5, and 7, respectively (Figure 3). EsxA and EsxB proteins were more abundant in lineage 4 than other lineages, while the EsxB protein showed lowest abundance in lineage 7 (Figure 4). Tuberculist was used to assign functional categories for the top 100 most abundant proteins for each lineage (Supplementary File S4). Lineages 5 and 7 exhibited higher abundance of proteins involved in intermediary metabolism and respiration than lineages 3 and 4. Proteins involved in lipid metabolism showed higher abundance in lineage 7 than other lineages. Cell wall and cell process-related proteins were more abundant in lineage 4 than in other lineages.

### Metabolic Proteins

Welch’s *t*-test revealed the proteins that were differentially abundant between the MTBC lineages (Supplementary Files S5–S10). The most distinct difference was observed between lineages 3 and 7 in which 221 proteins were differentially expressed; 138 were more abundant and 83 were less abundant (Supplementary File S5). The second highest difference was observed between lineages 4 and 7 that showed 177 differentially expressed proteins of which 97 were abundant upregulated and 80 less abundant (Supplementary File S6). Less difference was observed between lineages 3 and 4 where among the 87 differentially abundant proteins, 49 were more abundant and 38 less abundant (Supplementary File S7).

ANOVA was used to identify the 539 differentially abundant proteins (Supplementary Files S11,S12) that were further analyzed by unsupervised HCA to yield eight clusters/categories of differentially abundant proteins. The largest four clusters comprised 170, 124, 76, and 55 proteins. The major functional categories associated with these clusters include DNA repair and recombination, lipid metabolism, energy metabolism, ESX-1 and ESX-3 secretion system (Supplementary File S11). Gene ontology analysis among the lineages was performed to predict biological effects. Pathway sub-clusters that were significantly enriched are listed in Supplementary File S11. A subset of differentially expressed proteins were assigned to functional categories, including intermediary metabolism and respiration (23.01%), conserved hypothetical proteins (20.9%), lipid metabolism (16.3%), and cell wall and cell processes-related (19.2%) (Supplementary File S11B).

**FIGURE 5:**
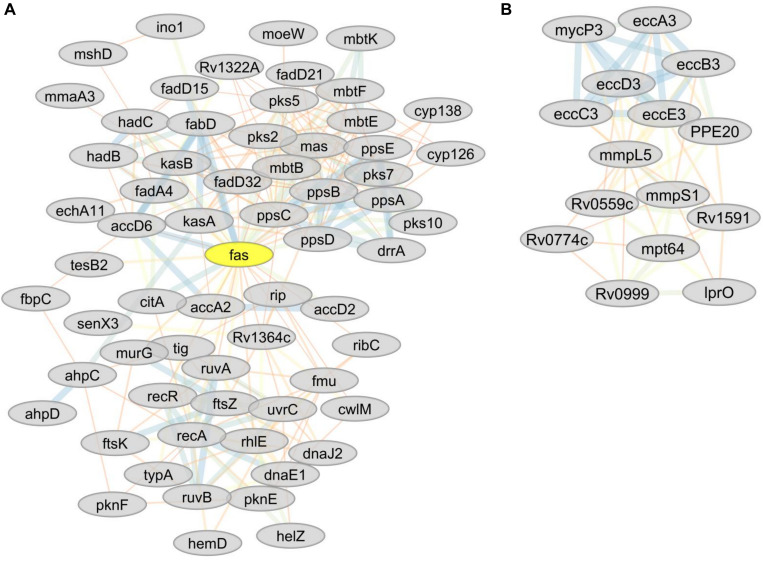
**(A)** Predicted sub-network interactions for differentially abundant proteins (RecA, FaS, FabD, PssD, and PssC) representing important protein hubs. **(B)** Predicted sub-network interactions for differentially abundant proteins involved in type VII ESX-3 secretion system (iron acquisition).

Analysis using the online STRING protein query database suggested potential protein-protein interactions involving 536 proteins resulting in 3021 putative interactions (e.g., protein network). Potentially important protein hubs involving RecA, FaS, FabD, PpsD, PpsC, EccB3, and EccC3 were identified (Figures 5A,B). Six network sub-clusters, playing potential roles in type VII ESX3-secretion, DNA repair, inorganic phosphate uptake, CRISPR-Cas system, lipid metabolism, bioenergetics, and energy metabolism were identified.

### MTBC Type VII Secretion Systems ESX-1 and ESX-3 Are Differentially Expressed

**FIGURE 6:**
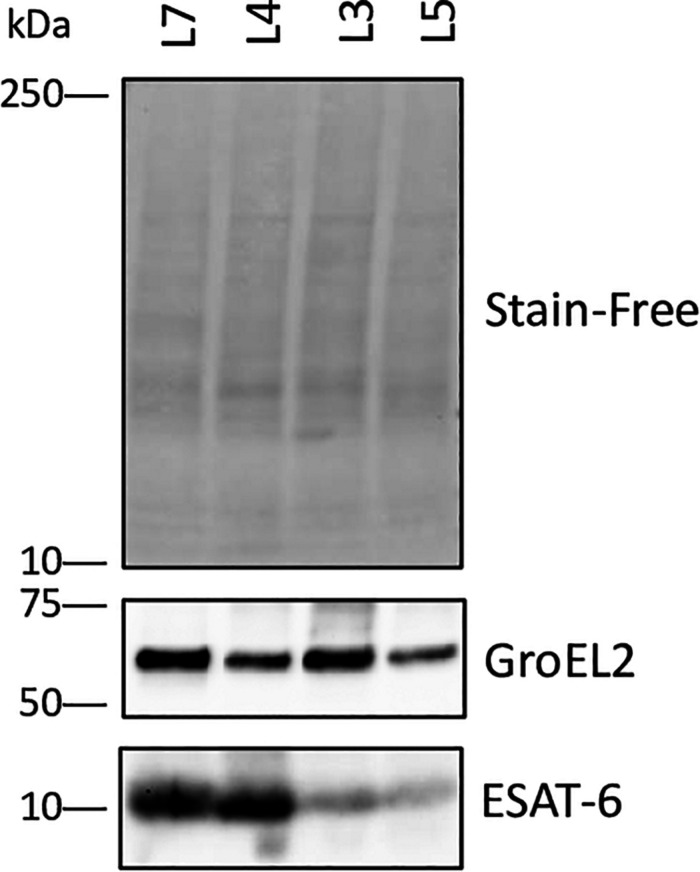
High expression of ESAT-6 in *M. tuberculosis* complex (MTBC) lineage 4. Immunoblotting demonstrated a very high abundance of ESAT-6 in lineage 4 strain H37Rv. Immunoblotting of 15 μg of total lysate from each strain using monoclonal antibody against ESAT-6 and GroEL2 confirmed the different amounts of ESAT-6 in protein samples from MTBC strains lineage 7 (L7), lineage 4 (L4), lineage 3 (L3) and lineage 5 (L5) detected by: Normalization control 1: The gel included a stain that stains tryptophan. When activated, the stain follows the protein over to the blot and can be used to detect the uniformity of transfer and that the amount of each sample loaded is similar (http://www.bio-rad.com/en-no/applications-technologies/stain-free-imaging-technology). This form of normalization control usually performs better than antibodies against housekeeping proteins for detection abnormalities. Normalization control 2: Immunoblotting with a monoclonal antibody against GroEl2. These data and the proteomics data (Supplementary file S13) indicated that the expression of GroEL2 was fairly even between the protein samples from strains of MTBC lineages 3, 4, 5, and 7.

The ESX-3 secretion component proteins including EccA3, EccB3, EccC3, EccD3, and EccE3 proteins were less abundant in lineage 7 than in other lineages (Table 1). The ESX-3 component proteins identified showed protein-protein interactions as shown in Figure 5B. The differentially expressed ESX-1 component proteins included EsxA, EsxB, EsxG, EccB, EspA, EspC, and EspD (Table 2). The EsxA protein was more abundant in lineage 4 than in lineages 3 and 5, as corroborated by immunoblotting, independent of biological or technical replicates (Figure 6 and Supplementary File S13). The EsxB proteins were less abundant in lineages 7 (Figure 6). The EspD and EspC proteins required for ESAT-6 secretion were more abundant in lineage 7 than in other lineages (Table 2).

**TABLE 1:** Differentially abundant proteins in the type VII ESX-3 secretion systems in MTBC lineages.

**Protein name**	**Gene symbol**	**Rv number**	**Less abundance lineage/s**	**Increased abundance lineage/s**
ESX-3 secretion system protein EccA3	*eccA3*	Rv0282	7	3, 4, 5
ESX-3 secretion system protein EccC3	*eccC3*	Rv0284	7	3, 4, 5
ESX-3 secretion system protein EccD3	*eccD3*	Rv0290	7	3, 4, 5
ESX-3 secretion system protein EccB3	*eccB3*	Rv0283	7	3, 4, 5
ESX-3 secretion system protein EccE3	*eccE3*	Rv0292	7	3, 4, 5
Membrane-anchored mycosin MycP	*mycp3*	Rv0291	7	3, 4, 5
Siderophore exporter MmpL5	*mmpl5*	Rv0675	7	3, 4, 5

**TABLE 2:** Differentially abundant proteins in the ESX-1 secretion system and other virulence related proteins in MTBC lineages.

**Protein name**	**Gene symbol**	**Rv number**	**Less abundance (lineage/s)**	**Increased abundance lineage/s)**
6 kDa early secretory antigenic target	*esxA*	Rv3873	3, 5	4, 7
ESAT-6-like protein EsxB	*esxB*	Rv3874	5, 7	3, 4
ESAT-6-like protein EsxG	*esxG*	Rv0287	5, 7	3, 4
ESX-1 secretion-associated protein EspA	*espA*	Rv3626c	3, 4	5, 7
ESX-1 secretion-associated protein EspC	*espC*	Rv3881c	3, 4, 5	7
7ESX-1 secretion-associated protein EspD	*espD*	Rv3614c	3, 4, 5	7
Putative antitoxin VapB10	*vapB10*	Rv1398c	5, 7	3, 4
Ribonuclease VapC15	*vapC10*	Rv1397c	5, 7	3, 4
PPE family protein PPE18	PPE18	Rv1196	7	3, 4, 5
PE family immunomodulator PE15	PE15	Rv1386	7	3, 4, 5
Uncharacterized PPE family protein PPE38	PPE38	Rv2352c	7	3, 4, 5
Cholesterol oxidase	*choD*	Rv3409c	7	3, 4, 5
Mce-family protein Mce1A	*mce1A*	Rv0169	3, 5, 7	4
Mce-family protein Mce1B	*mce1B*	Rv0170	3, 5, 7	4
Mce-family protein Mce1D	*mce1D*	Rv0172	3, 5, 7	4
Mce-family protein Mce1F	*Mce1F*	Rv0174	3, 5, 7	4
Immunogenic protein MPT64	*mpt64*	Rv1980C	7	3, 4, 5

Other virulence-related proteins including the mammalian cell entry family proteins (Mce1F, Mce1A, Mce1B and Mce1D) were more abundant in lineage 4. The anti-apoptotic component protein, Mpt64, and the type II toxin-antitoxin (TA) system (VapB10 and VapC10) were less abundant in lineage 7 (Table 2). Data on clusters 511, 515 and 528 suggest differential expression of type VII ESX-3 and ESX-1 secretion system/virulence-related proteins and other proteins (Supplementary File S11).

### DNA Repair and Recombination

**FIGURE 7:**
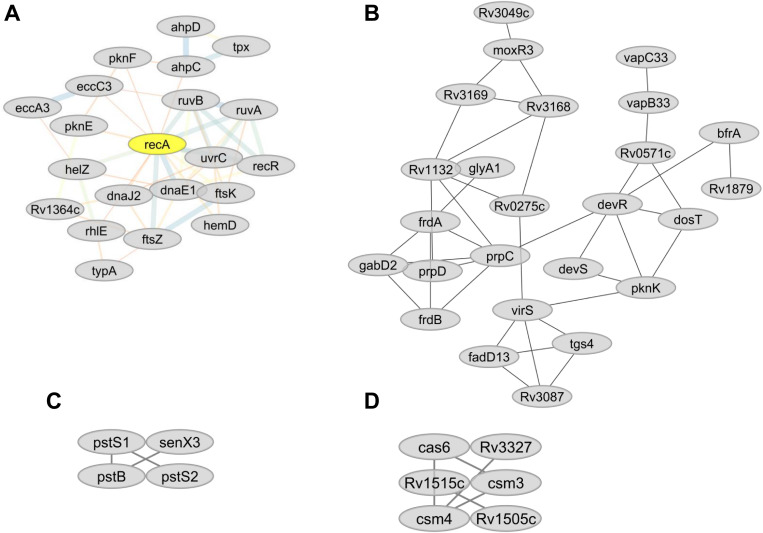
**(A)** Predicted sub-network interactions for differentially abundant proteins involved in DNA repair. **(B)** Protein sub-network interaction among proteins involved in energy metabolism. **(C)** Predicted sub-network interaction for differentially abundant proteins involved in inorganic phosphate uptake. **(D)** Predicted sub-network interaction for differentially abundant proteins involved in CRISPR-Cas systems.

Proteins that play essential roles in DNA recombination and repair (RecA, RuvA, RuvB, RecR, and UvrC) (Dos Vultos et al., 2009) were less abundant in lineage 7 than other lineages (Table 3). Low abundance in lineage 7 included DnaE1 (DNA replication proofreading), FtsK (DNA conjugation, DNA unwinding), NrdF2 (deoxyribonucleotide biosynthesis), DnaJ2 (heat shock, hyperosmotic shock), Rv0043c, Rv0795, NucS and CspA (DNA binding), and NadA, NadB, and NadC (DNA synthesis precursors). The majority of DNA repair proteins form protein–protein interactions and form a hub with RecA (Figure 7A). Cluster 530 also includes many DNA repair proteins (Supplementary File S11).

**TABLE 3:** Differentially abundant proteins involved in the DNA repair and Pst systems in MTBC lineages.

**Protein name**	**Gene ID**	**Rv number**	**Less abundance (lineage/s)**	**Increased abundance (lineage/s)**
Protein RecA	*recA*	Rv2737c	7	3, 4, 5
Holliday junction ATP-dependent DNA helicase RuvA	*ruvA*	Rv2593c	7	3, 4, 5
Holliday junction ATP-dependent DNA helicase RuvB	*ruvB*	Rv2592c	7	3, 4, 5
Recombination protein RecR	*recR*	Rv3715c	7	3, 4, 5
UvrABC system protein C	*uvrC*	Rv1420	7	3, 4, 5
DNA polymerase III subunit alpha	*dnaE1*	Rv1547	7	3, 4, 5
DNA translocase FtsK	*ftsk*	Rv2748c	7	3, 4, 5
Putative helicase HelZ	*helZ*	Rv2101	7	3, 4, 5
Ribonucleoside-diphosphate reductase subunit beta nrdF2	*nrdF2*	Rv3048c	7	3, 4, 5
Chaperone protein DnaJ 2	*dnaJ2*	Rv2373c	7	3, 4, 5
Uncharacterized HTH-type transcriptional regulator Rv0043c	*Rv0043c*	Rv0043c	7	3, 4, 5
Insertion element IS6110 uncharacterized 12.0 kDa protein	*Rv0795*	Rv0795	7	3, 4, 5
Phosphate-binding protein PstS 2	*pstS2*	Rv0932c	7	3, 4, 5
Phosphate-binding protein PstS 1	*pstS1*	Rv0934	7	3, 4, 5
Phosphate import ATP-binding protein PstB 1	*pstsB1*	Rv0933	7	3, 4, 5

### Lipid Metabolism

Proteins involved in mycolic acid and phthiocerol dimycocerosate (PDIM) biosynthesis including FaS, FadD15, EchA11, HadB, MmaA3, and Pks13 were less abundant in lineage 7 (Table 4). Fas-II component proteins (KasA, KasB, and HadB), carboxylases (AccA2, AccD2, and AccD6) (Duan et al., 2014), and RV2006 involved in trehalose biosynthesis (Kapopoulou et al., 2011) were less abundant in lineages 4 and 7. Fas forms an important interaction hub (Figure 5A).

**TABLE 4:** Differentially abundant proteins involved in lipid metabolism in MTBC lineages.

**Protein name**	**Gene symbol**	**Rv number**	**Less abundance (lineage/s)**	**Increased abundance lineage/s)**
Fatty acid synthase Fas	*fas*	Rv2524c	7	3, 4, 5
Long-chain-fatty-acid–CoA ligase FadD15	*fasD15*	Rv2187	7	3, 4, 5
Probable enoyl-CoA hydratase EchA11	*EchA11*	Rv1141c	7	3, 4, 5
(3R)-Hydroxyacyl-ACP dehydratase subunit HadB	*hadB*	Rv0636	7	3, 4, 5
Polyketide synthase Pks13	*pks13*	Rv3800c	7	3, 4, 5
Methoxy mycolic acid synthase MmaA3	*mmaA3*	Rv0643c	7	3, 4, 5
3-Oxoacyl-[acyl-carrier-protein] synthase 1 KasA	*kasA*	Rv2245	7	3, 4, 5
3-Oxoacyl-[acyl-carrier-protein] synthase 2	*ksaB*	Rv2246	7	3, 4, 5
Probable acetyl-/propionyl-coenzyme A carboxylase alpha chain (Alpha subunit) AccA2	*accA2*	Rv0973c	4, 7	3, 5
Probable acetyl-/propionyl-CoA carboxylase (Beta subunit) AccD2	*accD2*	Rv0974c	4, 7	3, 5
Propionyl-CoA carboxylase beta chain 6 AccD6	*accD6*	Rv2247	4, 7	3, 5
Malonyl CoA-acyl carrier protein transacylase	*fadD*	Rv0166	5, 7	3, 4
Long-chain-fatty-acid–AMP ligase FadD32	*fadD32*	fadD32	5, 7	3, 4
Galactofuranosyltransferase GlfT2	*GlfT2*	Rv3808c	5, 7	3, 4
Phthiocerol synthesis polyketide synthase type I PpsA	*ppsA*	Rv2931	4, 7	3,5
Phthiocerol synthesis polyketide synthase type I PpsB	*ppsB*	Rv2932	4, 7	3, 5
Phenolpthiocerol synthesis type-I polyketide synthase PpsC	*ppsC*	Rv2933	4, 7	3, 5
Doxorubicin resistance ATP-binding protein DrrA	*drrA*	Rv2936	4, 7	3, 5
Phthiocerol synthesis polyketide synthase type I PpsD	*ppsD*	Rv2934	4, 7	3, 5
Phthiocerol synthesis polyketide synthase type I PpsE	*ppsE*	Rv2935	4, 7	3, 5
Steroid 3-ketoacyl-CoA thiolase	*fadA5*	Rv3546	4, 7	3, 5
Polyketide synthase Pks7	*Pks7*	Rv1661	4, 7	3, 5
Long-chain-fatty-acid–AMP ligase FadD26	*fadD26*	Rv2930	4, 7	3, 5
Polyketide synthase-like Pks10	Pks10	Rv1660	5, 7	3, 4
Phthioceranic/hydroxyphthioceranic acid synthase	*Pks2*	Rv3825c	5, 7	3, 4
Phthiotriol/phenolphthiotriol dimycocerosates methyltransferase	*Rv2952*	Rv2952	7	3,4, 5
Putative phthiocerol dimycocerosate transporter LppX	*lppx*	Rv2945c	3,7	4, 5
Mycocerosic acid synthase-like polyketide synthase	*pks5*	Rv1527c	7	3, 4
Arabinofuranosyltransferase AftB	*aftb*	Rv3805c	3, 4, 7	5
Putative diacyglycerol *O*-acyltransferase Rv2285	*Rv2285*	Rv2285	7	3, 4, 5
Peptidoglycan endopeptidase RipA	*ripA*	Rv1477	4	3, 7
Peptidoglycan endopeptidase RipB	*ripB*	Rv1478	7	3, 4, 5
Probable penicillin-binding protein DacB1	*dacB1*	Rv3330	7	3, 4, 5
Phosphoglucosamine mutase	*glmM*	Rv3441c	7	3, 4, 5
Rhamnosyl *O*-methyltransferase	*Rv2959c*	Rv2959c	3, 7	4, 5
Uncharacterized glycosyl hydrolase Rv2006	*Rv2006*	Rv2006	4, 7	3, 5
Putative inactive phenolphthiocerol synthesis polyketide synthase type I Pks1	*pks1*	Rv2946c	3, 4, 7	5
Steroid C26-monooxygenase	*cyp125*	Rv3545c	3,4, 7	5
Long-chain-fatty-acid–AMP ligase FadD30	*fadD30*	Rv0404	3, 4, 7	5
Acyl-CoA dehydrogenase FadE28	*fadE28*	Rv3544c	3, 4, 7	5
Probable NADPH dependent 2,4-dienoyl-CoA reductase FadH	*fadh*	Rv1175c	5, 7	3, 4
UDP-*N*-acetylmuramoyl-tripeptide–D-alanyl-D-alanine ligase	*murF*	Rv2157c	3, 5, 7	4
UDP-*N*-acetylmuramoylalanine–D-glutamate ligase	*murD*	Rv2155c	5, 7	3, 4
UDP-*N*-acetylmuramoyl-L-alanyl-D-glutamate–2,6-diaminopimelate ligase	*murE*	Rv2158c	3, 5, 7	4
Isoniazid-induced protein IniC	*iniC*	Rv0343	5, 7	3, 4
Isoniazid-induced protein IniA	*iniA*	Rv0342	5, 7	3, 4

Proteins required for PDIM/phenolglycolipid (PGL) biosynthesis including PpsA, PpsB, PpsD, PpsE, MaS, DrrA, Pks7, FadA5, and FadD26 (Domenech and Reed, 2009) were less abundant in lineages 4 and 7. MaS and Rv2952 proteins involved in PGL synthesis (Domenech and Reed, 2009) were less abundant in lineage 7. FadE28 and Cyp125 that catalyze the activation of long-chain fatty acids, LppX that is involved in PDIM translocation, and AftB responsible for arabinogalactan biosynthesis (Kapopoulou et al., 2011) were more abundant in lineage 5. Rv2959c that catalyzes the O-methylation linked to the phenolic group of glycosylated PGL (Simeone et al., 2013) was more abundant in lineages 4 and 5.

Among proteins involved in cell wall peptidoglycan biosynthesis, MurE, MurF, FadD5, and RipA were more abundant in lineage 4 than other lineages. In addition, IniC and IniA proteins that are targets for peptidoglycan biosynthesis inhibitors were more abundant in lineages 3 and 4 than other lineages (Table 4). The heatmaps corresponding to clusters 530 and 515 depict the differential abundance of lipid biosynthesis-related proteins and other proteins (Supplementary File S11).

### Energy Metabolism

Several proteins responsible for energy metabolism (glycolysis, TCA cycle, and oxidative phosphorylation) were differentially expressed (Table 5). PgI is involved in glycolysis (Kapopoulou et al., 2011), PfkA required for gluconeogenesis, the AdhA which catalyzes the reversible oxidation of ethanol for the reduction of NAD, and LldD1 involved in the conversion of lactate into pyruvate (Kapopoulou et al., 2011) were more abundant in lineage 7 than other lineages.

**TABLE 5:** Differentially abundant proteins involved in energy metabolism in MTBC lineages.

**Protein name**	**Gene symbol**	**Rv number**	**Less abundance (lineage/s)**	**Increased abundance lineage/s)**
Glucose-6-phosphate isomerase Pgi	*pgI*	Rv 0946c	3, 4, 5	7
6-Phosphofructokinase PfkA	*pfkA*	Rv3010c	3, 4, 5	7
Probable alcohol dehydrogenase AdhA	*adhA*	Rv1862	3, 4, 5	7
Possible L-lactate dehydrogenase (cytochrome) LldD1	*LldD1*	Rv0694	3, 4, 5	7
Putative citrate synthase II CitA	*citA*	Rv0889c	7	3, 4, 5
Isocitrate lyase Icl	*Icl1*	Rv0467	4, 7	3, 5
6-Phosphogluconate dehydrogenase Gnd1	*gnD1*	Rv1844c	5, 7	5, 7
6-Phosphogluconate dehydrogenase, decarboxylating Gnd2	*gnD2*	Rv1122	5, 7	5, 7
Glucose-6-phosphate 1-dehydrogenase Zwf2 (G6PD)	*Zwf2*	Rv1447c	3, 4, 5	7
Fumarate reductase flavoprotein subunit	*frdA*	Rv1552	3, 5	4, 7
Fumarate reductase iron-sulfur subunit	*frdB*	Rv1553	3, 5	4, 7
Putative succinate-semialdehyde dehydrogenase 2	*gabD2*	Rv1731	3, 5	4, 7
Citrate synthase 1	*gltA2*	Rv0896	5	3, 4, 7
Succinate dehydrogenase 2 membrane subunit SdhC	*sdhC*	Rv3316	5	3, 4, 7
Succinate dehydrogenase flavoprotein subunit	*sdhA*	Rv3318	5	3, 4, 7
Probable succinate dehydrogenase SdhB	*sdhB*	sdhB	5	3, 4, 7
2-Methylcitrate synthase	*prpC*	Rv1131	3, 4, 5	7
2-Methylcitrate dehydratase	*prpD*	Rv1130	3, 4, 5	7
Probable NADH dehydrogenase Ndh	*ndH*	Rv1854C	4, 7	3, 5
NADH dehydrogenase-like protein Rv1812c	*nadH*	Rv1812c	7	3, 4, 5
Probable NADPH dependent 2,4-dienoyl-CoA reductase FadH	*fadH*	RV1812c	5, 7	3, 4
Quinolinate synthase A	*nadA*	Rv1594	7	3, 4, 5
L-Aspartate oxidase	*nadB*	Rv1595	7	3, 4, 5
Nicotinate-nucleotide pyrophosphorylase	*nadC*	Rv1596	7	3, 4, 5
Isocitrate lyase 2	*aceaB*	Rv1916	4	3, 5, 7
Putative isocitrate lyase subunit A	*aceaA*	Rv1915	4	3, 5, 7

Among the proteins involved in the oxidative direction of the TCA cycle, CitA in lineage 7 and Icl in lineages 4 and 7 were less abundant than other lineages. Proteins involved in alternative pathways of the TCA cycle were differentially abundant, among which the PrpD and PrpC proteins required for methyl citrate cycle pathway, and the GnD1 and GnD2 (Kapopoulou et al., 2011) proteins involved in the pentose phosphate pathway were more abundant in lineages 5 and 7. Zwf2 was more abundant in lineage 7 than in other lineages.

The fumarate reductases (FrdA and FrdB) that play vital roles in the maintenance of an energized membrane during hypoxia (Watanabe et al., 2011) and GabD2, an essential protein for generating succinate from succinic semialdehyde (SSA), were more abundant in lineages 4 and 7. The interaction of these proteins is depicted in Figure 7B. In contrast, lineage 5 showed low expression of GltA2 and the succinate dehydrogenase enzymes (SdhA, SdhB, SdhD) compared to other lineages.

Among the proteins involved in oxidative phosphorylation (electron transport chain), lineages 4 and 7 showed increased abundance of Ndh. FadH was less abundant in both lineages 5 and 7, and Rv1812c was less abundant in lineage 7 than other lineages. A number of highly expressed proteins involved in energy metabolism showed direct protein-protein interaction (Figure 7B). The heatmap in cluster 511, 513, 523, and 528 shows the differential abundance of various proteins among which proteins involved in energy metabolism are also listed (Supplementary File S11).

### Stress Responses Proteins

Several proteins that have critical roles in protection against metabolic stress were differentially regulated (Table 6). MshD, Tpx, AhpC and AhpD were less abundant in lineage 7 than in other lineages. The serine/threonine-protein kinases (PknE, PknF, Rv0043, SenX3) involved in regulatory functions were less abundant in lineage 7 than other lineages. All proteins involved in metabolic stress response are shown in the heatmaps corresponding to clusters 530 together with other proteins (Supplementary File S11).

**TABLE 6:** Differentially abundant metabolic stress response and regulatory proteins in MTBC lineages.

**Protein name**	**Gene symbol**	**Rv number**	**Less abundance (lineage/s)**	**Increased abundance lineage/s)**
Alkyl hydroperoxide reductase C	*ahpC*	Rv2428	4, 5, 7	3
Alkyl hydroperoxide reductase AhpD	*ahpD*	Rv2429	7	3, 4, 5
Mycothiol acetyltransferase	*mshD*	Rv0819	7	3, 4, 5
Thiol peroxidase	*tpX*	Rv1932	7	3, 4, 5
Serine/threonine-protein kinase PknE	*pknE*	Rv1743	7	3, 4, 5
Serine/threonine-protein kinase PknF	*pkmF*	Rv1746	7	3, 4, 5
Uncharacterized HTH-type transcriptional regulator Rv0043c	Rv0043c	Rv0043c	7	3,4, 5
Sensor-like histidine kinase senX3	*senX3*	Rv0490	7	3, 4, 5
DNA-binding transcriptional activator DevR/DosR	*devR/DosR*	Rv3133c	3,7	5, 7
Oxygen sensor histidine kinase response regulator DosT	*dosT*	Rv2027c	3, 4, 5	7
Oxygen sensor histidine kinase response regulator DevS/DosS	*devS*	Rv3132c	4, 5, 7	3

### ABC Transporters

Ammonium bicarbonate transporter proteins PstS1, PstS2, and PstB that are essential for the high-affinity capture of periplasmic inorganic phosphate (Pi) in Mtb (Peirs et al., 2005) and synthesis of DNA molecules were less abundant in lineage 7 (Table 3). Proteins involved in copper homeostasis including CtpV, CtpG multi-copper oxidase (MmcO) and CsoR were more abundant in lineage 5 than other lineages. The protein-protein interaction network of the Pst system proteins is depicted in Figure 7C.

### CRISPR-Cas Systems

Mtb lineage 7 harbors an abundance of CRISPR-Cas systems, and some of these components were expressed in an MTBC lineage-specific manner. Csm3 and Csm4 were more abundant in lineage 7, while the expression of the Cas6 was upregulated in lineages 4 and 7. Cas10, Csm3, Csm4, Cas6, and Cas10 proteins were also more abundant in lineage 7. Csm3, Csm4, Csm5, Cas6 exhibit pathways interaction with each other (Figure 7D), and are found in clusters 511, 528, and 529 (Supplementary File S10).

**FIGURE 8:**
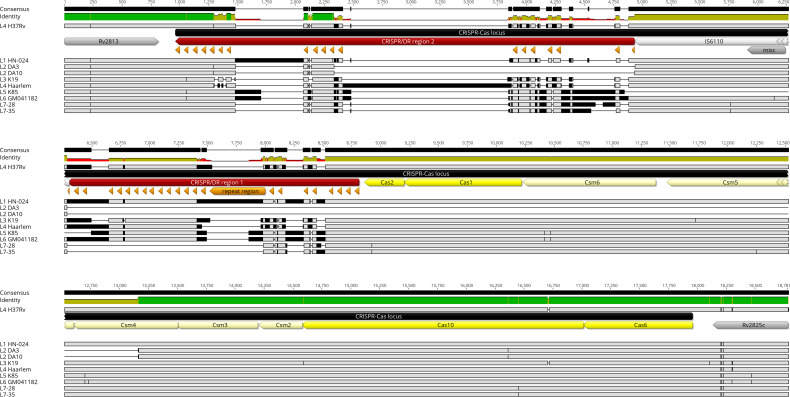
Shows multiple genome alignment of all lineages using Geneious Prime software. The analysis focuses on CRISPR-CAS locus in the following order: CRISPR-Cas region 2, IS6110 (mobile element), CRISPR-Cas region 1, Cas2, Cas1, Csm6, Csm5, Csm4, Csm3, Csm2, Cas10, and Cas6. The Rv2813 and Rv2825c that flank the CRISPR-Cas locus are included in the alignment as reference points. As shown, lineage 2 (ID no. DA 3 and DA 10) have deletion of most the CRISPR repeats. Both Cas 1 and 2, and Csm6 - Csm4 (half of Csm4 is deleted). Cas 10 and Cas 6 seem to be highly conserved among the lineages. SNPs have been observed (black horizontal lines). The size of SNPs in Lineage 7 strains (ID L28 and L35) seems smaller than in other lineages.

Multiple genome alignment analysis of the CRISPR-Cas locus revealed the presence of single nucleotide polymorphisms (SNPs) between the lineages. Notably, the number of SNPs observed in Csm3, Csm4, Csm5, and Cas6 in the two lineage 7 strains was reduced as compared to other lineages (Figure 8).

## Discussion

This study documented MTBC lineage-specific protein abundances in clinical strains of lineages 3, 4, 5, and 7. The coverage of the predicted MTBC proteome (77%) is similar to that reported elsewhere (Bespyatykh et al., 2016; Jhingan et al., 2016; Yimer et al., 2016; Schubert et al., 2018).

The Esx-3 secretion system is essential for siderophore-mediated iron uptake and is critical for MTBC isolates growth *in vitro* (Houben et al., 2014). Mycobactin and carboxymycobactin are the main siderophore molecules in MTBC that are required for iron uptake (De Voss et al., 1999). Biosynthesis of mycobactin and carboxymycobactin requires MbtB, MbtE and MbtF proteins, and we observed that MbtE and MbtF were less abundant in lineage 7. In addition, IdeR, MmpL5 and MycP3 are involved in iron homeostasis, siderophore biosynthesis and secretion, and *in vitro* growth, respectively (De Voss et al., 1999; Pandey and Rodriguez, 2014; Siegrist et al., 2014). The decreased abundance of proteins involved in ESX-3 secretion suggests reduced uptake of iron, which may affect growth and virulence in lineage 7.

Several proteins involved in the ESX-1 secretion system were differentially abundant in a lineage-specific manner. MTBC requires ESAT6 and EsxB for full virulence (Forrellad et al., 2013; Houben et al., 2014). ESAT6 and EsxB facilitate the translocation of Mtb from the phagosome into the host cell cytoplasm (van der Wel et al., 2007; Houben et al., 2012; Simeone et al., 2012; Jamwal et al., 2016). ESAT6 inhibits antigen-presentation and TLR signaling, IL-12 production and macrophage-dependent apoptosis (Pathak et al., 2007; Mtb with defects in secretion of EsxA and/or EsxB are not translocated into the cytosol and show decreased host cell lysis and cell-to-cell invasion (Guinn et al., 2004; Houben et al., 2012). EsxG inhibits phagosome maturation (Mehra et al., 2013). The increased abundance of EsxA, EsxB and EsxG in lineages 4 and 3 contribute to enhanced growth and increased virulence. These lineages are widely distributed globally (Coscolla and Gagneux, 2014).

Efficient ESAT-6 secretion in Mtb is dependent on abundant expression of the EspA, EspB, EspC, and EspD proteins (Fortune et al., 2005; MacGurn et al., 2005). EspD stabilizes the cellular levels of EspA and EspC. The EspC is a potent antigen in both active and latent TB infection. The immunodominance of EspC is similar to ESAT6 as has been proposed to be a promising TB vaccine candidate (Millington et al., 2011). The high abundance of EspA, EspC, and EspD in lineages 5 and 7 may suggest a compensatory mechanism for the relatively low abundance of EsxA and EsxB observed in these two lineages.

The mammalian cell entry (Mce) proteins are surface-exposed secreted proteins and crucial virulence factors (Arruda et al., 1993). The abundance of Mce1 proteins in Mtb lineage 4 may contribute to the high virulence of this lineage. Mtb has anti-apoptotic capacities to counteract host microcidal activity. The abundance of PknE and Mpt64 were low in lineage 7. Mpt64 inhibits macrophage apoptosis *in vitro* (Wang et al., 2014). PknE prevents apoptosis by interfering with host signaling pathways (Jayakumar et al., 2008).

Several proteins that have critical roles in lipid biosynthesis (mycolic acid and PDIM) were differentially expressed. Mycolic acids are vital components of the MTBC cell wall contributing to inherent drug resistance, growth and virulence (Duan et al., 2014; Marrakchi et al., 2014). The synthesis of mycolic acids requires type I and type II fatty acid synthase (FAS). Fas-II involves KasA, KasB, HadABC, Rv0635, Rv0636, Rv0637, Rv0033, and the InhA proteins (Duan et al., 2014). Among the Fas-II proteins, KasA, KasB, InhA, HadB, Rv0033, and Pks13 were less abundant in lineages 4 and 7. KasA catalyzes the early steps of fatty acid elongation reaction, and KasB extends the elongation products to complete the length of fatty acid chains (Schiebel et al., 2013). Deletion of the *kasB* gene resulted in colony morphology defect, loss of acid fastness and subclinical latent TB in mice (Bhatt et al., 2007). InhA is required in the final step of fatty acid extension (Gurvitz et al., 2008). Pks13 catalyzes the condensation reaction to produce mycolic acids precursors (Duan et al., 2014). Mycolic acid biosynthesis also requires building blocks (acylCoAs) that are synthesized from carboxylases. The MTBC genome contains *accA1, accA2 and accA3* biotin carboxylase subunits and six carboxyltransferase subunits (*accD1–accD6*). *accD6* is part of the FAS-II complex genetic locus (37). Lineage 7 exhibited a lower abundance of AccA2, AccD2 and AccD6. RV2006, which is less abundant in lineage 7, is used in trehalose biosynthesis. Trehalose is a major free sugar molecule which is an integral part of cell wall glycolipids (Murphy et al., 2005). Trehalose is crucial in mycolic acid transport during cell wall biogenesis.

The MTBC cell wall is composed of complex lipids such as PDIM in addition to mycolic acids. Proteins including the Pks1, Pk5, Pks7, FadD26, FadD28, and Pks10 that showed decreased abundance in lineage 7 are involved in PDIM biosynthesis (Azad et al., 1997). One study reported that the *pks1 and pks10* mutants displayed deficient PDIM production and attenuated virulence in mice (Forrellad et al., 2013). The *fadD28* mutant showed defect in PDIM biosynthesis (Forrellad et al., 2013).

Peptidoglycan is an essential component of the bacterial cell wall, which is crucial in maintaining cell shape and strength (Kouidmi et al., 2014). Mur ligases (MurD, MurE, and MurF) and RipA play significant roles in peptidoglycan biosynthesis. DacB1, which is less abundant in lineage 7, is involved in crosslinking of cell wall peptidoglycan strands (Goodsmith et al., 2015). The cytoplasmic biogenesis of fatty acids is coordinated with a set of steps in charge of leading them to their destination out of the cell. The proper synthesis and localization of cell wall is compromised if there is any derangement in the formation and exporting steps of fatty acids. Given that several proteins involved in the essential steps of lipid biosynthesis were less abundant in lineage 7, the possibility of defects in cell wall formation and growth in this lineage is very high.

Mtb uses homologous recombination (HR) to repair DNA damage and several proteins are essential (Singh et al., 2016). Lineage 7 exhibited a low abundance of important DNA repair components including UvrC, RecA, RuvA, RuvB, RecR, DnaE1, and HelZ proteins. Our previous study showed non-synonymous SNPs in genes in lineage 7 strains involved in DNA metabolism such as *nadD* and *dnaB* (helicase) (Yimer et al., 2016). Notably, three of the five proteins involved in NAD+ cofactor synthesis (Kurnasov et al., 2003), i.e., NadA, NadB, and NadC, were less abundant in lineage 7. NAD+ cofactor starvation is detrimental in the tubercle bacilli (Boshoff et al., 2008). As DNA repair and recombination are expected to play crucial roles in the adaptative potential, evolution and pathogenicity of Mtb, it is tempting to speculate that the decreased abundance of these essential DNA repair/recombination components in lineage 7 may imply less efficient DNA repair mechanism or slow growth.

The CRISPR-Cas genome editing system protects cells against phage infection (Barrangou and Marraffini, 2014). The CRISPR-derived RNA (crRNA) is responsible for directing the invader-degrading Cas complex to the invader (Hille and Charpentier, 2016). crRNA synthesis requires Cas6, Cas10, Csm2, Csm3, Csm4, and Csm5. Mutations in Cas-Csm genes have been shown to affect immunity in *Staphylococcus aureus* (Hatoum-Aslan et al., 2014). Csm3 acts as a ruler and measures the size of incoming crRNA maturation within the Cas10-Csm complex in *S. epidermidis* (Hatoum-Aslan et al., 2013). Cas10 displays rapid conformational fluctuations on foreign RNA targets, but is locked in a static configuration on self RNA, highlighting the central role of self versus non-self discrimination and target specificity (Wang et al., 2019). The lineage 7 strains exhibited an increased abundance of proteins involved in the CRISPR-Cas system. This observation is supported by the multiple-genome alignment analysis of the CRISPR-Cas locus which revealed very low number of SNPs in the genomes of lineage 7 strains than in other lineages. Mutations in the genes encoding CRISPR-Cas systems or the lack of CRISPR-Cas systems generally contribute to drug resistance (Wei et al., 2019). Deletion of Cas1 was found to reduce the sensitivity to anti-tuberculosis drugs by decreasing the persistence during treatment (Wei et al., 2019). Our previous study revealed that strains of lineages 3 and 4 exhibited a higher propensity for resistance to anti-TB drugs than lineage 7 strains (Yimer et al., 2015). The increased abundance of the Csm3, Csm4, Cas6 and Cas10 in lineage 7 may protect against phage infection and drug resistance. These possibilities warrant further study.

Metabolic stress adaptation is essential for MTBC survival. A number of proteins involved in metabolic stress responses were differentially abundant in a lineage-specific manner. TpX, which is less abundant in lineage 7, catalyzes the reduction of hydroperoxides and peroxynitrite (Rho et al., 2006). A *tpx-*mutant Mtb strain showed impaired replication in macrophages (Hu and Coates, 2009). MshD involved in mycothiol (MSH) biosynthesis has a similar function to glutathione (Newton et al., 2008). Mutations in *mshD* resulted in MSH production defect (Newton et al., 2005; Venkatesan et al., 2016). An *ahpC* mutant Mtb showed decreased survival in macrophages (Master et al., 2002). These proteins were less abundant in lineage 7 and may result in an inefficient metabolic response to oxidative stress, which warrants further functional study.

Proteins involved in cellular respiration were differentially abundant in a lineage-specific manner. The TCA pathway supplies NADH for use in oxidative phosphorylation (Cavalcanti et al., 2014). CitA and IcL proteins, which are less abundant in lineage 7, are essential to catalyze the biosynthesis of citrate and isocitrate to produce NADH. Among the enzymes involved in oxidative phosphorylation, lineage 7 showed less abundance of key enzymes (Ndh, Rv1812C and FadH) (Cook et al., 2014). Ndh is one of the major oxidoreductases in mycobacteria (Iqbal et al., 2018) and has a catalytic function crucial for reducing the flight of electrons to oxygen during transfer from NADH to menaquinone. Decreased NADH abundance causes starvation and disruption of cellular redox homeostasis and results in cell death (Iqbal et al., 2018). The question is therefore how does lineage 7 in particular maintain fitness for survival given the decreased abundance of several key enzymes involved in the oxidative direction of TCA cycle and electron transport chain? The TCA cycle is dynamic. When Mtb encounters hostile environmental conditions such as hypoxia, the bacteria shift the oxidative TCA cycle to a reductive direction and start to secrete succinic acid to complete the respiratory cycle. This is facilitated by succinate dehydrogenase (SDH) that forms complex II of the respiratory chain. Complex II of the respiratory chain is composed of SdhA, SdhB, SdhC, and SdhD proteins (Cook et al., 2014; Iqbal et al., 2018), of which SdhA and SdhB were more abundant in lineage 7. These enzymes oxidize succinate to fumarate in the cytoplasm. Fumarate reductase (FRD), a “paralog” of SDH, catalyzes the reverse reaction. We found an increased abundance of FrdA and FrdB in lineages 4 and 7 that may indicate a shift in metabolic pathway in these two lineages. It has been shown FrdA was more abundant under hypoxic conditions in Mtb (Sharma and Tyagi, 2016) and that TCA was repressed in dormant culturable Mtb cells (Converse et al., 2009). Lineage 7 also exhibited a higher abundance of several proteins that are involved in alternative pathways of the TCA cycle. This may be considered a compensatory mechanism for generating energy to maintain basic physiological functions of Mtb lineage 7 cells and to survive harsh environmental conditions.

Lineages 3 and 4 demonstrated a higher abundance of IniC and IniA that play key roles in the development of resistance to both isoniazid and ethambutol (Colangeli et al., 2005; Almeida Da Silva and Palomino, 2011). Lineage 4 has been frequently associated with drug resistance (Fenner et al., 2012; Tessema et al., 2013; Gygli et al., 2017). In addition, lineages 3 and 4 showed higher level of drug tolerance than other Mtb lineages in Ethiopia (Yimer et al., 2015).

## Conclusion

This study presents novel findings regarding differential protein abundance/protein expression in Mtb lineage 7 versus the other clinically relevant MTBC lineages 3, 4, and 5. The reduced abundance of proteins involved in type VII ESX-3 and ESX-1 secretion systems, TCA cycle and oxidative phosphorylation, lipid metabolism and DNA repair in lineage 7 may contribute to the slow growth and less virulent phenotype, which corroborates a previous report (Yimer et al., 2017). The increased abundance of proteins involved in the CRISPR-Cas system in lineage 7 may enable protection against phage infection and drug resistance. Given the increased abundance of several proteins in Mtb lineage 7 that are involved in alternative energy metabolism pathways, we hypothesize that increased respiratory plasticity may contribute to the ability of this lineage to survive adverse growth conditions possibly through increased periods of dormancy.

The increased abundance of proteins involved in type VII ESX-3 and ESX-1 secretion systems, DNA repair, drug resistance and lipid metabolism may contribute to the high virulence and increased fitness for survival of lineage 4, which is consistent with the wide global distribution of this lineage. Lineage 3, prevalent in central Asia and East Africa, showed increased abundance of proteins involved in lipid metabolism, ESX-3 secretion system, the TCA cycle and other metabolic pathways associated with increased fitness and survival capacity. The results of this study may provide an insight into the phenotypic differences among clinically important MTBC lineages. However, additional studies using models for *in vivo* infection are warranted to confirm these findings.

## Data Availability Statement

The datasets presented in this study can be found in online repositories. The names of the repository/repositories and accession number(s) can be found in the article.

## Ethics Statement

The studies involving human participants were reviewed and approved by Regional Committee for Medical Research Ethics in Eastern Norway (REK Øst) and the Ethiopian Science and Technology Ministry in Addis Ababa, Ethiopia. The patients/participants provided their written informed consent to participate in this study.

## Author Contributions

SAY and TT conceived the study and study design. SAY conducted the field work and collected lineages 3, 4, and 7 isolates in Ethiopia. AGB and EDZ performed specimen cultivation. SK and EDZ performed sample inactivation and lysate preparation for MS analysis. HH performed immunoblotting. SK performed MQ analysis. TR ran MS analysis. SK and SAY performed proteome bioinformatics and statistical analysis. AGB performed cytoscape. TL ran Geneious Prime for sequence analysis. SAY, TT, and SK evaluated and interpreted the data and drafted the manuscript. All authors edited and approved the final manuscript.

## Conflict of Interest

The authors declare that the research was conducted in the absence of any commercial or financial relationships that could be construed as a potential conflict of interest.

## Footnotes

1 https://www.geneious.com

2 http://www.interactivenn.net/

3 http://string-db.org/

4 https://cytoscape.org/index.html

## Supplementary Material

FILE S1 A multi-histogram demonstrates the distribution of protein spectra detected across all the experimental replicates. The frequency distribution of the log_2_-transformed LFQ intensities has a unimodal shape with the typical “bell shaped” normal distribution. This distribution remained the same after the dataset was imputed for missing values. Proteins exhibiting low abundance and/or poorly ionizing peptides are highlighted by the red color.

FILE S2 Depicts the complete list of protein groups identified in all lineages.

FILE S3 Shows the iBAQ intensities in all proteomes that covered a dynamic range of four orders of magnitude between the most- and least abundant proteins.

FILE S4 The top 100 most abundant proteins assigned by their functional categories using Tuberculist (Kapopoulou et al., 2011).

FILE S5 Protein abundance comparison using Welch’s *t*-test between the MTBC lineages: Protein abundance difference between lineages 3 and 7.

FILE S6 Protein abundance comparison using Welch’s *t*-test between the MTBC lineages: Protein abundance comparison between lineages 4 and 7.

FILE S7 Protein abundance comparison using Welch’s *t*-test between the MTBC lineages: Protein abundance comparison between lineages 3 and 4.

FILE S8 Protein abundance comparison using Welch’s *t*-test between the MTBC lineages: Protein abundance comparison between lineages 5 and 7.

FILE S9 Protein abundance comparison using Welch’s *t*-test between the MTBC lineages: Protein abundance comparison between lineages 4 and 5.

FILE S10 Protein abundance comparison using Welch’s *t*-test between the MTBC lineages: Protein abundance comparison between lineages 3 and 5.

FILE S11 List of the 539 differentially abundant proteins based on ANOVA analysis. The DA protein groups were further subjected to unsupervised hierarchical cluster analysis resulting in eight clustered protein groups.

FILE S12 Gene ontology analysis among the MTBC lineages 3, 4, 5, 7. Pathway sub-clusters that were significantly enriched are listed. The list also includes significant enrichment analysis between the lineages: 5 and 7, 3 and 7, and 4 and 7.

FILE S13 Detailed experimental procedure for immunoblotting.
