# Supplementary material for: Lineage-Specific Proteomic Signatures in the Mycobacterium tuberculosis Complex Reveal Differential Abundance of Proteins Involved in Virulence, DNA Repair, CRISPR-Cas, Bioenergetics and Lipid Metabolism
Source: Front Microbiol. 2020 Sep 22;11:550760. doi: 10.3389/fmicb.2020.550760 (PMC7536270; doi:10.3389/fmicb.2020.550760)
Supplement: FILE S1 — A multi-histogram demonstrates the distribution of protein spectra detected across all the experimental replicates. The frequency distribution of the log2-transformed LFQ intensities has a unimodal shape with the typical “bell shaped” normal distribution. This distribution remained the same after the dataset was imputed for missing values. Proteins exhibiting low abundance and/or poorly ionizing peptides are highlighted by the red color. [file Data_Sheet_1.pdf]

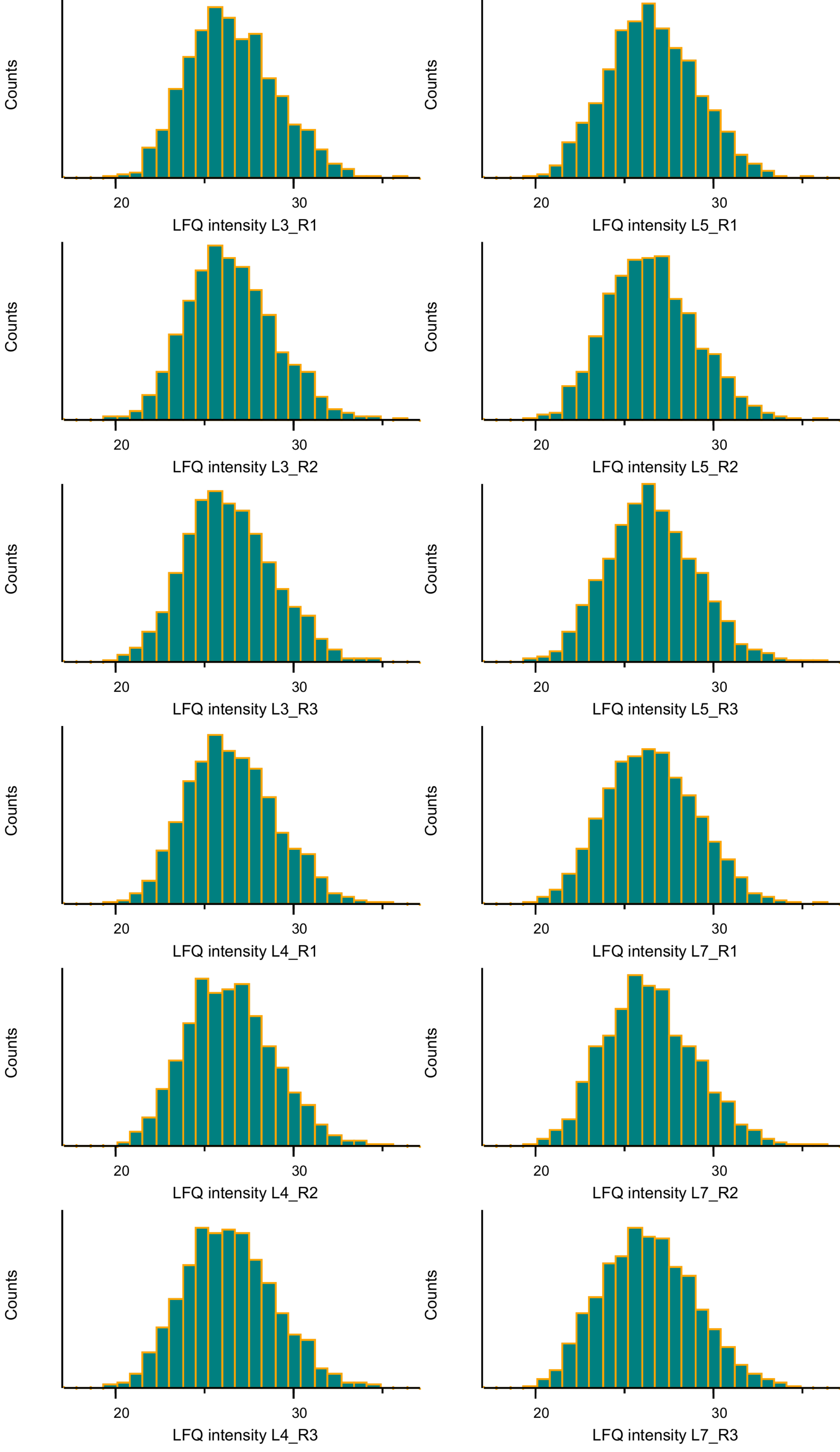
Counts
20
30
LFQ intensity L3_R1
LFQ intensity L5_R1
LFQ intensity L3_R2
LFQ intensity L5_R2
LFQ intensity L3_R3
LFQ intensity L5_R3
LFQ intensity L4_R1
LFQ intensity L7_R1
LFQ intensity L4_R2
LFQ intensity L7_R2
LFQ intensity L4_R3
LFQ intensity L7_R3
