# Supplementary material for: Lineage-Specific Proteomic Signatures in the Mycobacterium tuberculosis Complex Reveal Differential Abundance of Proteins Involved in Virulence, DNA Repair, CRISPR-Cas, Bioenergetics and Lipid Metabolism
Source: Front Microbiol. 2020 Sep 22;11:550760. doi: 10.3389/fmicb.2020.550760 (PMC7536270; doi:10.3389/fmicb.2020.550760)

Experimental procedure for western GroE2 and ESAT-6

Western blot of 15µg of total lysate from each strain using monoclonal antibody against ESAT-6, confirming the different amounts of ESAT-6 in the different strains. Samples L7, L4, L3 and L5, full names on next page.

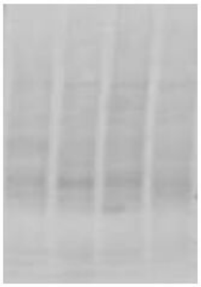
Stain-Free image of blot: 2020-07-09 22hr 17min 27sec_stainfree_blot2_crop.tif

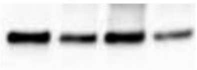
GroEL2 blot: 2020-07-10 14hr 08min 45sec_GroEL2_blot2_crop.tif

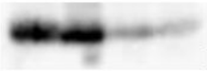
ESAT6 blot: 2020-07-10 15hr 31min 11sec_ESAT6_blot2_crop.tif

From pptx-file

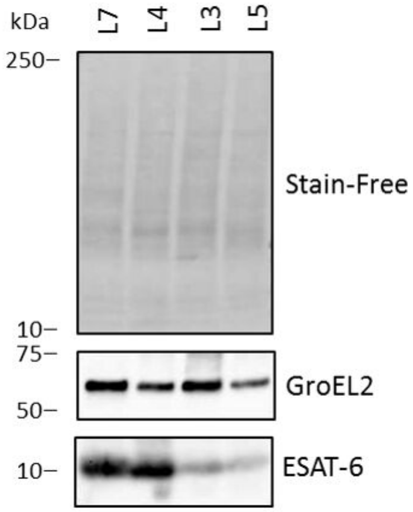

Procedural details

Gel: Biorad Mini-protean TGX Stain-Free TGX gel, 4-20%, 12 well, 20 µl

Samples heated 5 min a 95C in sample buffer with B-me before running.

Gel run 100V for 1h

Membrane: Trans blot turbo Transfer pack 0.2µm PVDF

Transfer: Trans blot turbo with TGX mini program.

Western buffer TBS-T: 10mM Tris pH=7.5, 150 mM NaCl, 0,05% TWEEN 20

Blocking: 8 hour TBS-T with 5% dry skimmed milk.

Primary antibody: ESAT-6 monoclonal antibody (11G4) from ThermoFisher Scientific. Diluted 1:1000 in TBS-T with 5% milk. 2 hours at RT

Primary antibody: GroEL2 mouse monoclonal antibody (BDI578) from OriGene. Diluted 1:1000 in TBS-T with 5% milk. 1 hour at RT

Secondary antibody: Santa cruz SC-516102 m-IgGκ BP-HRP. Diluted 1:5000 in TBS-T with 5% milk, 1 hour

Detection: Reagent: ECL™ Prime Western Blotting System. Instrument: Chemidoc Touch with luminescence detection.

Washing: 3x10 min in TBS-T inbetween all steps and 6x in the last step detection.

Samples: The same set of samples as used for MS. Values measured previously did show not very good normalization on stainfree last time. Remeasured all samples in triplicates and redid normalization. Looks a bit better.

Many of original triplicates are empty. Choose 2 pr strain and performed western twice on each sample. Linage 5 have only 1 sample left.

Normalization control 1: The gel includes a stain that stains tryptophan. When activated the stain follows the protein over to the blot and can be used to see the uniformity of transfer and that the amount of each sample loaded is similar. Usually performs better than antibodies against housekeeping proteins for detection abnormalities. http://www.bio-rad.com/en-no/applications-technologies/stain-free-imaging-technology

Normalization control 2: GroEl2. Suggested normalization control from reviewer. Data from Shewit indicates that GroEL2 expression is fairly even between the samples from different strains.

L3_R1	L3_R2	L3_R3	L4_R1	L4_R2	L4_R3	L5_R1	L5_R2	L5_R3	L7_R1	L7_R2	L7_R3	ANOVA Significant	Gene IDs	RV numbers	Protein name
31.8406	30.8106	31.8175	33.8377	32.8193	33.0129	30.7158	29.3106	31.2281	33.0355	32.3724	31.9639	+	esxA	Rv3875	6kDaearlysecretoryantigenictarget
32.4521	33.7418	32.9579	34.9643	35.0665	34.5858	33.1436	32.699	32.9058	31.0986	32.0254	31.8199	+	esxB	Rv3874	ESAT-6-likeproteinEsxB
27.9841	27.572	27.161	28.8839	27.9068	27.7387	25.8928	24.7967	25.5955	24.9304	25.7163	24.3028	+	esxG	Rv0287	ESAT-6-likeproteinEsxG
29.6148	30.573	30.3252	31.4877	30.5495	30.7103	30.5413	28.6618	30.3368	28.4885	28.8753	28.6161	+	esxK	Rv1197	ESAT-6-likeproteinEsxK
27.7125	28.7588	29.0845	29.1172	28.9641	29.2348	29.8633	30.1518	30.3464	27.7096	28.1557	28.0868	+	esxN	Rv1793	ESAT-6-likeproteinEsxN
30.549	28.4295	31.0802	31.5861	31.6704	31.4056	22.3105	25.9042	25.8639	28.2046	29.4717	29.363	+	esxO	Rv2346c	ESAT-6-likeproteinEsxO
32.7361	32.5372	32.5027	32.233	32.4687	32.5158	32.5691	33.0725	33.0217	33.0056	33.7943	33.6425		groEL1	Rv3417c	60kDachaperonin1
35.9857	35.732	35.7815	35.1439	35.565	35.7813	34.9638	35.8048	35.6871	35.9977	36.277	36.4687		groEL2	Rv0440	60kDachaperonin2

Blot cut in half so that high molecular weight probed with GroEL2 antibody and low molecular weight probed with ESAT6 antibody

A) Stain-free image of the blot confirming that similar amounts of each sample is loaded on the gel and transferred to the blot.
B) Western blot of 15µg of total lysate from each strain using monoclonal antibody against ESAT-6, confirming the different amounts of ESAT-6 in the different strains.
C) Western blot of 15µg of total lysate from each strain using monoclonal antibody against GroEL2 confirming similar loading.

Sample Naming

Strain	Short name
SOL.101 (22.12.14) 1	L3-1 EMPTY
SOL.101 (22.12.14) 2	L3-2
SOL.101 (22.12.14) 3	L3-3
ESP 471.1 (22.12.14) 1	L4-1
ESP 471.1 (22.12.14) 2	L4-2
ESP 471.1 (22.12.14) 3	L4-3
M. Africanum (22.12.14) 1	L5-1 EMPTY
M. Africanum (22.12.14) 2	L5-2
M. Africanum (22.12.14) 3	L5-3 EMPTY
L7 #28 (according to Alemayehu) 1	L7-1
L7 #28 (according to Alemayehu) 2	L7-2
L7 #28 (according to Alemayehu) 3	L7-3

Gels and blots:

Lane	Gel1 and Gel2 identical
1	Biorad Precision Plus Dual color 5 µl
2	Biorad unstained Precision plus 20 µl
3	L7-3
4	L4-1
5	L3-2
6	L5-3
7	Biorad unstained Precision plus 20 µl
8	L7-2
9	L4-2
10	L3-3
11	L5-3
12	Biorad unstained Precision plus 20 µl

2020-07-09 21hr 40min 10sec_stainfree_gel_1

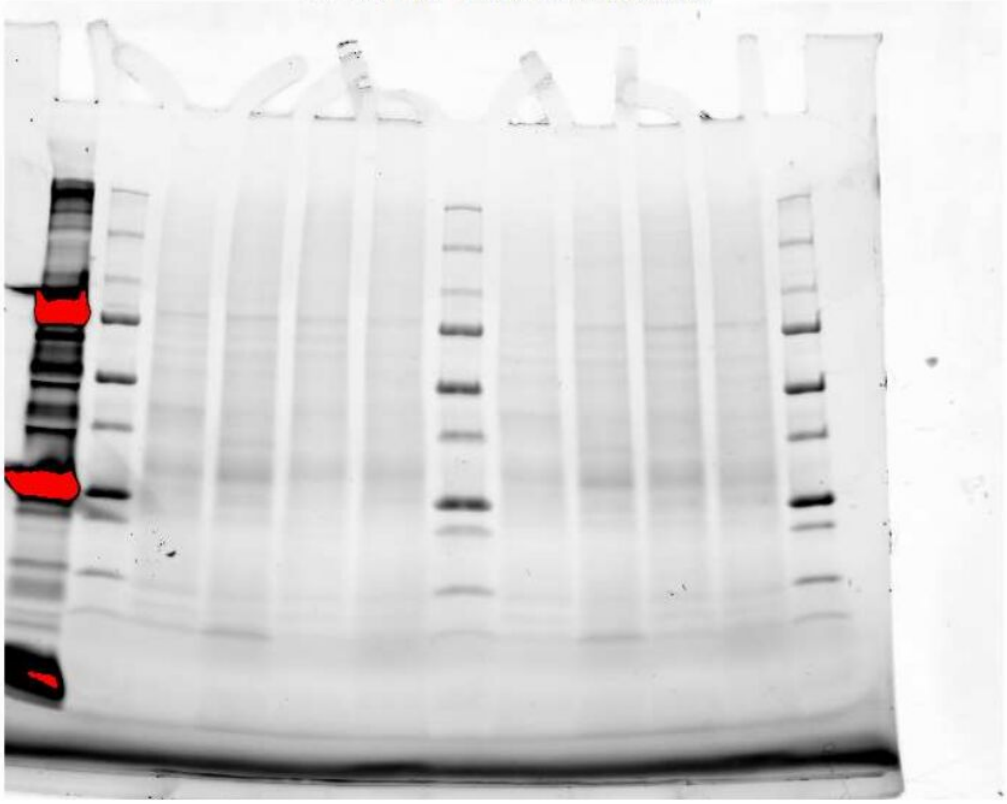

2020-07-09 21hr 55min 06sec_stainfree_gel_2

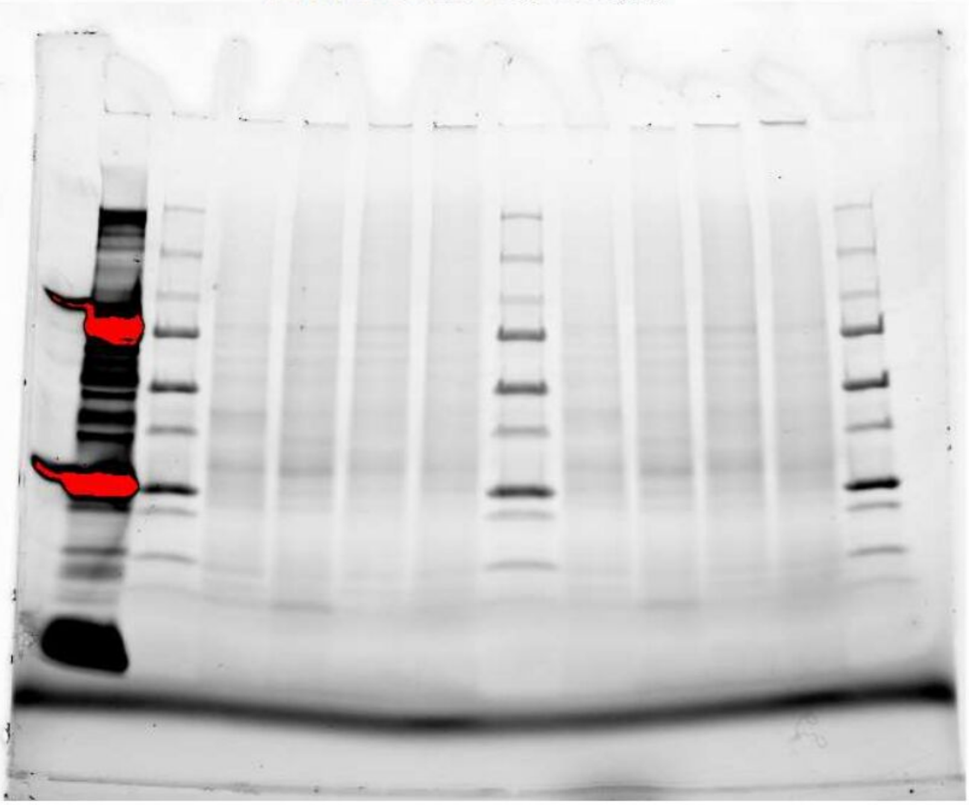

2020-07-09 22hr 08min 15sec_stainfree_blot1

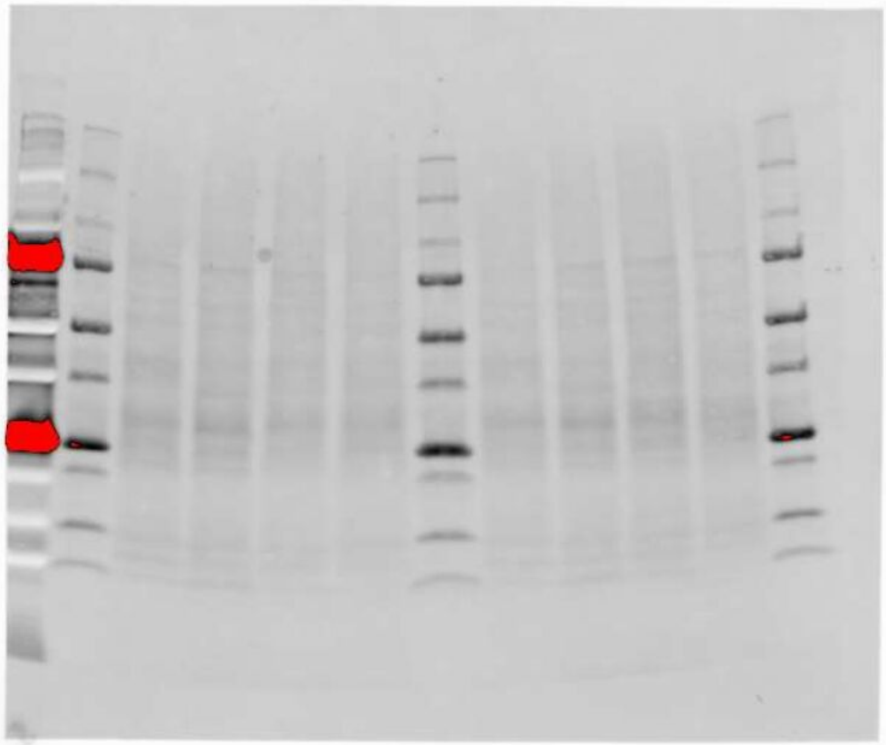

2020-07-09 22hr 17min 27sec_stainfree_blot2

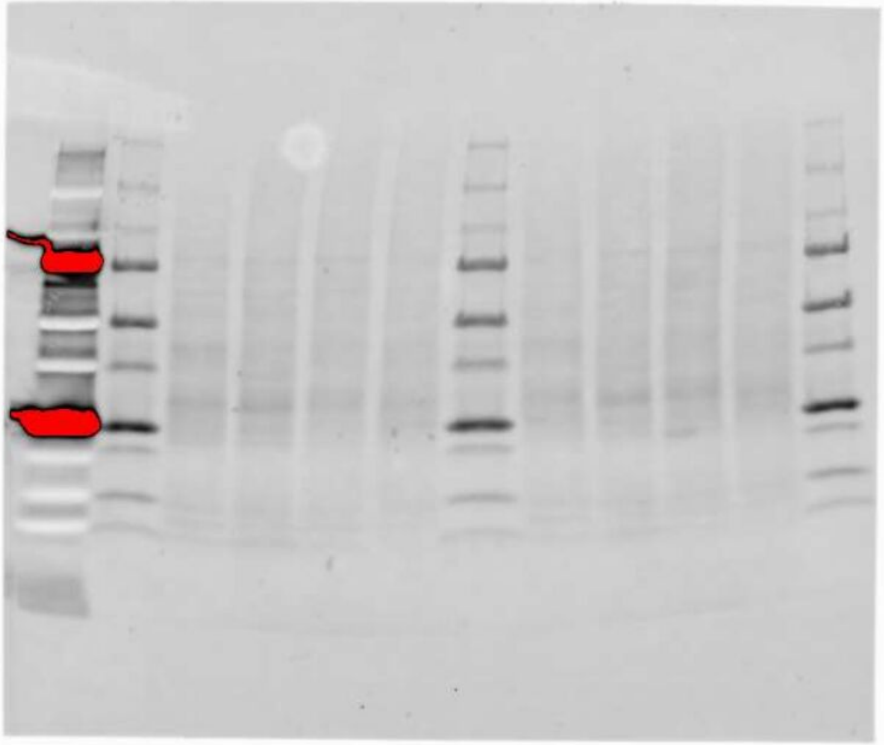

2020-07-10 13hr 52min 32sec_GroEL2_blot1

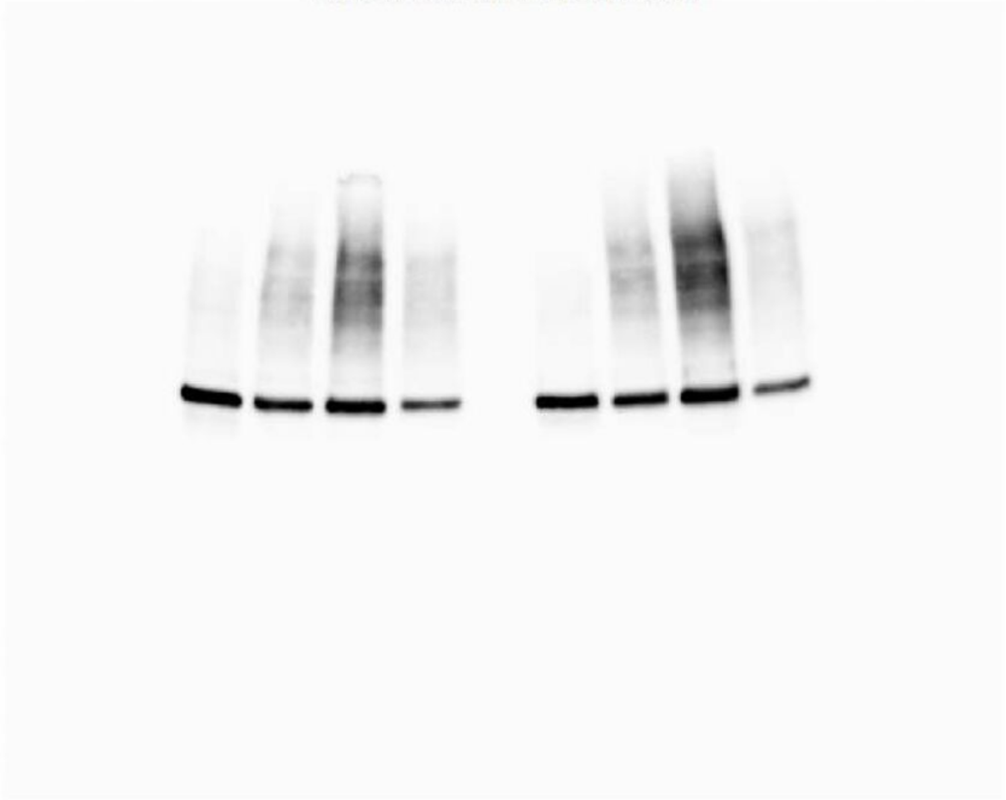

2020-07-10 15hr 01min 33sec_ESAT6_blot1

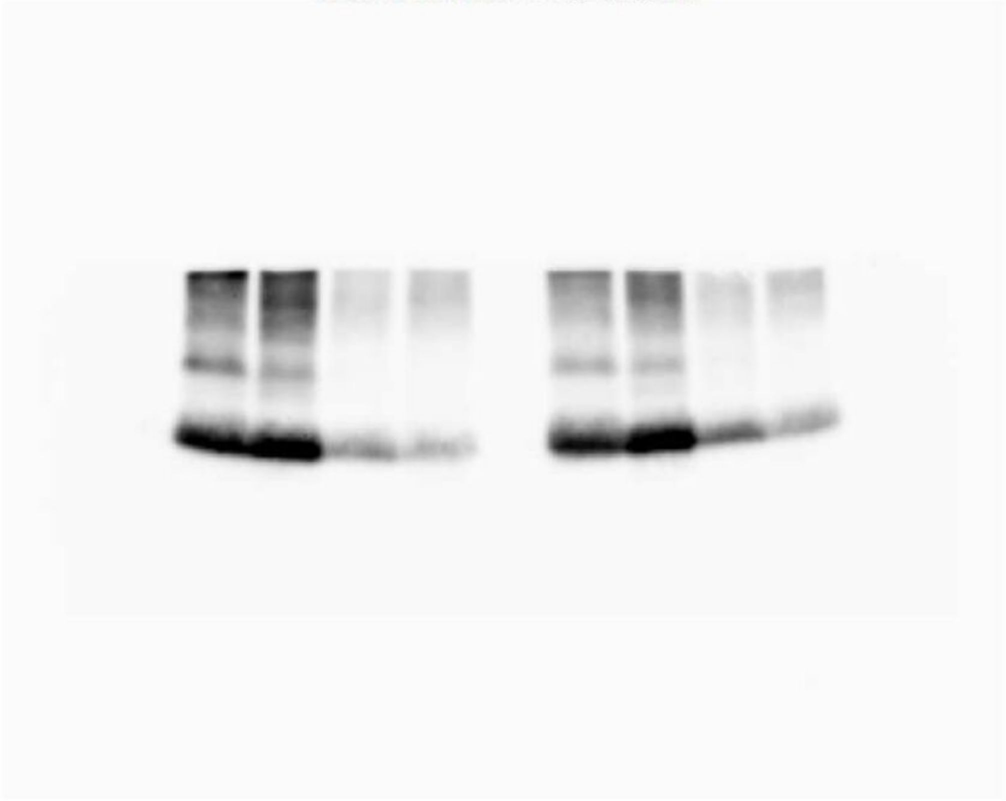

2020-07-10 14hr 08min 45sec_GroEL2_blot2

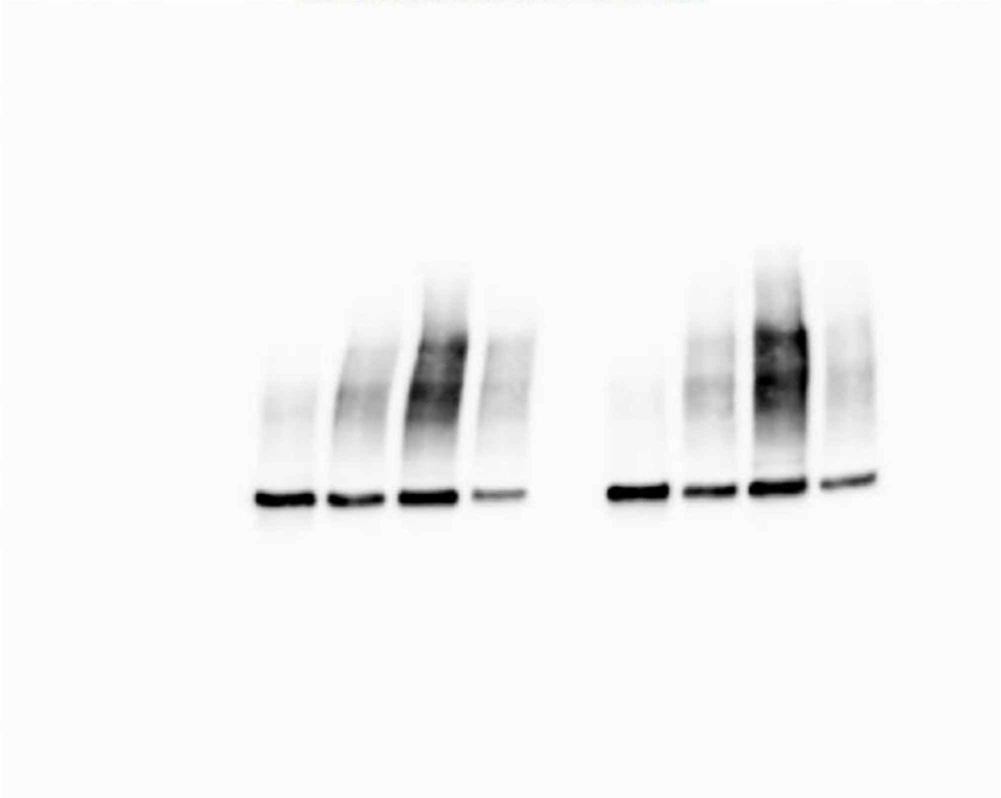

2020-07-10 15hr 31min 11sec_ESAT6_blot2

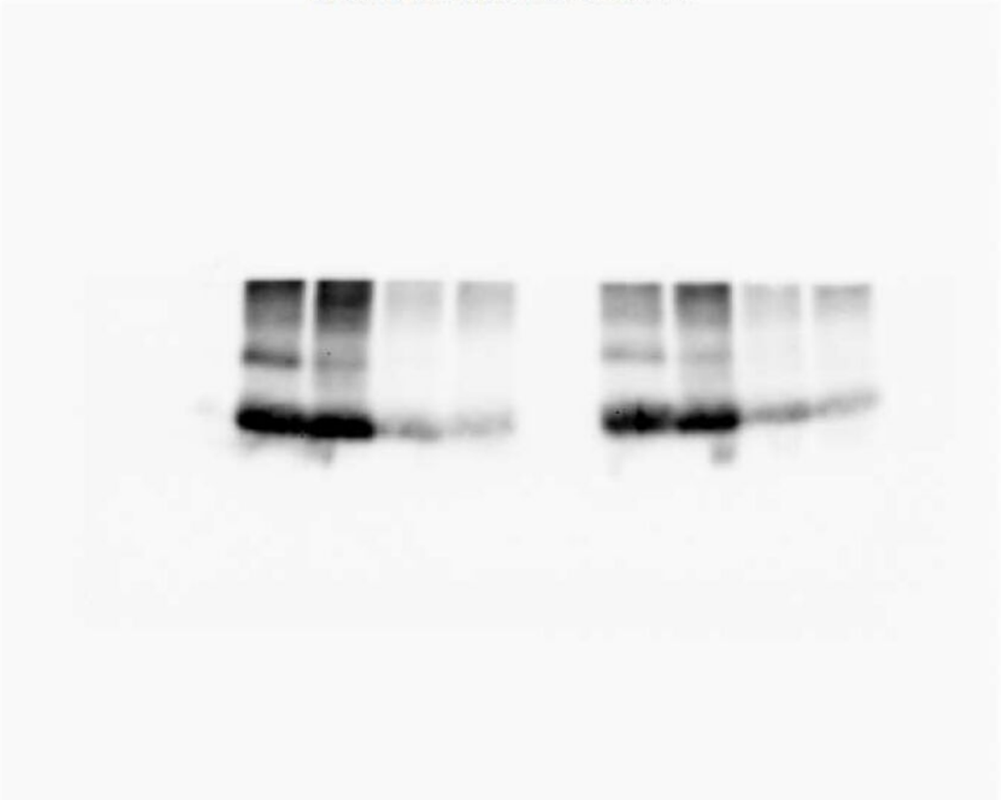

Supplement: FILE S13 — Detailed experimental procedure for immunoblotting. [file Data_Sheet_13.pdf]
